# CCC- and WASH-mediated endosomal sorting of LDLR is required for normal clearance of circulating LDL

**DOI:** 10.1038/ncomms10961

**Published:** 2016-03-11

**Authors:** Paulina Bartuzi, Daniel D. Billadeau, Robert Favier, Shunxing Rong, Daphne Dekker, Alina Fedoseienko, Hille Fieten, Melinde Wijers, Johannes H. Levels, Nicolette Huijkman, Niels Kloosterhuis, Henk van der Molen, Gemma Brufau, Albert K. Groen, Alison M. Elliott, Jan Albert Kuivenhoven, Barbara Plecko, Gernot Grangl, Julie McGaughran, Jay D. Horton, Ezra Burstein, Marten H. Hofker, Bart van de Sluis

**Affiliations:** 1 Department of Pediatrics, Molecular Genetics Section, University of Groningen, University Medical Center Groningen, Antonius Deusinglaan 1, 9713 AV Groningen, The Netherlands; 2 Department of Immunology, Mayo Clinic College of Medicine, Mayo Clinic, 200 First Street Southwest, Rochester, Minnesota 55905, USA; 3 Department of Biochemistry and Molecular Biology, Mayo Clinic College of Medicine, Mayo Clinic, 200 First Street Southwest, Rochester, Minnesota 55905, USA; 4 Department of Clinical Sciences of Companion Animals, Faculty of Veterinary Medicine, Utrecht University, Yalelaan 108, 3584 CM Utrecht, The Netherlands; 5 Department of Molecular Genetics, University of Texas Southwestern Medical Center, 5323 Harry Hines Boulevard, Dallas, Texas 75390, USA; 6 Department of Vascular and Experimental Vascular Medicine, Academic Medical Center, University of Amsterdam, Meibergdreef 9, 1105 AZ Amsterdam, The Netherlands; 7 Department of Pediatrics, University of Groningen, University Medical Center Groningen, Antonius Deusinglaan 1, 9713 AV Groningen, The Netherlands; 8 Department of Medical Genetics, University of British Columbia, 4500 Oak Street, Vancouver, British Columbia, Canada V6H 3N1; 9 Department of Pediatrics and Adolescence Medicine, Division of Child Neurology, Medical University Graz, Auenbruggerplatz 2, A-8036 Graz, Austria; 10 Division of Child Neurology, University Children's Hospital, Steinwiesstrasse 75, CH-8032 Zurich, Switzerland; 11 Genetic Health Queensland at the Royal Brisbane and Women's Hospital, Herston, University of Queensland, Herston, Brisbane, Queensland 4029, Australia; 12 School of Medicine, University of Queensland, Herston, Brisbane, Queensland 4029, Australia; 13 Department of Internal Medicine, University of Texas Southwestern Medical Center, 5323 Harry Hines Boulevard, Dallas, Texas 75390, USA; 14 Department of Molecular Biology, University of Texas Southwestern Medical Center, 5323 Harry Hines Boulevard, Dallas, Texas 75390, USA

## Abstract

The low-density lipoprotein receptor (LDLR) plays a pivotal role in clearing atherogenic circulating low-density lipoprotein (LDL) cholesterol. Here we show that the COMMD/CCDC22/CCDC93 (CCC) and the Wiskott–Aldrich syndrome protein and SCAR homologue (WASH) complexes are both crucial for endosomal sorting of LDLR and for its function. We find that patients with X-linked intellectual disability caused by mutations in *CCDC22* are hypercholesterolaemic, and that COMMD1-deficient dogs and liver-specific *Commd1* knockout mice have elevated plasma LDL cholesterol levels. Furthermore, *Commd1* depletion results in mislocalization of LDLR, accompanied by decreased LDL uptake. Increased total plasma cholesterol levels are also seen in hepatic COMMD9-deficient mice. Inactivation of the CCC-associated WASH complex causes LDLR mislocalization, increased lysosomal degradation of LDLR and impaired LDL uptake. Furthermore, a mutation in the WASH component *KIAA0196* (strumpellin) is associated with hypercholesterolaemia in humans. Altogether, this study provides valuable insights into the mechanisms regulating cholesterol homeostasis and LDLR trafficking.

## Abstract (web summary)

Low density lipoprotein receptor (LDLR) is crucial for cholesterol homeostasis. Here, the authors show that components of the CCC-protein complex, CCDC22 and COMMD1, facilitate the endosomal sorting of LDLR and that mutations in these genes cause hypercholesterolemia in dogs and mice, providing new insights into regulation of cholesterol homeostasis.

## Graphical abstract

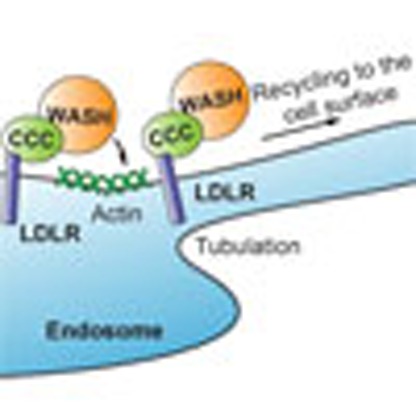

Various transmembrane proteins in the endosomal compartment depend on the Wiskott–Aldrich syndrome protein and SCAR homologue (WASH) complex to find their appropriate destination in the cell^1^,^2^,^3^. This pentameric protein complex, which consists of WASH1, FAM21, strumpellin, KIAA1033 (also known as SWIP) and CCDC53, is recruited to endosomes by the retromer complex^2^,^4^,^5^,^6^. Retromer is formed by the vacuolar protein-sorting (VPS) proteins VPS26, VPS29 and VPS35, and in concert with sorting nexins, it selectively mediates endosomal cargo sorting into recycling and retrieval pathways^7^. Recently, the COMMD/CCDC22/CCDC93 (CCC) complex has been identified to interact and colocalize with retromer and the WASH complex^2^,^8^,^9^. The CCC complex consists of the copper metabolism MURR1 domain-containing (COMMD) proteins, coiled-coil domain-containing protein 22 (CCDC22), coiled-coil domain-containing protein 93 (CCDC93) and C16orf62 (ref. 8). Among the 10 COMMD proteins^10^, COMMD1, a gene product that is mutated in Bedlington terriers affected by a hepatic copper storage disorder resulting in copper toxicosis^11^, was shown to regulate in concert with the WASH complex the recycling of the copper transporter ATP7A (ref. 8). In this same study, we reported that X-linked intellectual disability (XLID) patients carrying a mutation in *CCDC22* (c.49A>p.T17A) also have aberrant copper homeostasis, as serum copper and serum ceruloplasmin levels are increased in these patients^8^.

Given the pleiotropic function of COMMD1 (ref. 12) and CCDC22 (refs 2, 8, 9, 13, 14), and the large number of membrane proteins sorted by the WASH complex^3^, it is expected that the CCC complex is involved with numerous physiological processes. Here we identify that the CCC complex regulates the level of circulating low-density lipoprotein (LDL) cholesterol by mediating the endosomal trafficking of the low-density lipoprotein receptor (LDLR). Mutations affecting the formation of the CCC complex cause hypercholesterolaemia in humans, dogs and mice. We further show that LDLR is an endosomal cargo of the CCC-associated WASH complex, and inactivation of this complex results also in LDLR mislocalization and impaired LDL uptake. This study provides novel insights into the molecular mechanism causing hypercholesterolaemia, and highlights the importance of CCC and WASH complexes in cholesterol homeostasis.

## Results

### *CCDC22* mutations are associated with hypercholesterolaemia

On further clinical analysis of a large XLID family affected with a *CCDC22* p.T17A mutation we discovered that these patients also have an increase in total plasma cholesterol and LDL cholesterol levels (Table 1 and Supplementary Fig. 1a), exceeding the 95th percentile corrected for age and gender^15^,^16^. One of the patients (V-2, 4 years of age) is too young, and plasma cholesterol levels are not informative in this case^17^. In another XLID family with a *CCDC22* mutation (p.Y557C) (ref. 18), we found that the circulating total cholesterol (TC) and LDL cholesterol of the two patients carrying the mutation were also above the 95th percentile (Table 1 and Supplementary Fig. 1b). These observations suggest that mutations in the CCC component *CCDC22* are causally related to hypercholesterolaemia.

### COMMD1 deficiency results in hypercholesterolaemia in dogs

**Figure 1:**
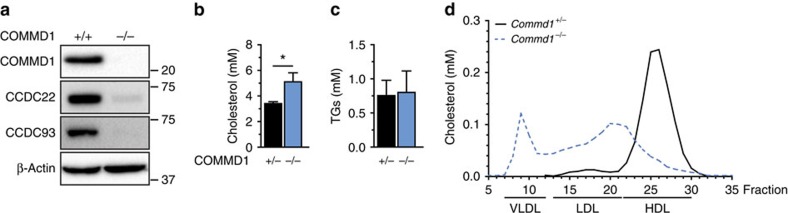
COMMD1-deficient dogs are hypercholesterolaemic. (**a**) Western blot analysis of COMMD1, CCDC22 and CCDC93 in livers from an unaffected dog (*COMMD1*^+/+^) and an affected dog homozygous for a loss-of-function mutation in *COMMD1* (*COMMD1*^*−/−*^). (**b**) Plasma TC and (**c**) TG levels of dogs heterozygous (+/−) (*n*=5) or homozygous (−/−) (*n*=4) for a *COMMD1* mutation (**d**) FPLC lipoprotein profile of *COMMD1*^*+/−*^ (*n*=5) and *COMMD1*^*−/−*^ dogs (*n*=4). Pooled plasma samples of each experimental group were separated using FPLC gel filtration. Fifty fractions were collected from each separation. TC content was determined and lipoprotein profile was plotted. The results are presented as mean±s.e.m., and significance was calculated relative to the control group by unpaired Student's *t*-test; *P<0.05.

Since *CCDC22* mutation (p.T17A) and COMMD1 inactivation both impairs the formation of a stable CCC complex^8^,^13^, we were prompted to investigate the plasma cholesterol levels in dogs with a *COMMD1* loss-of-function mutation^11^,^19^. In addition to the expected copper accumulation in the liver of these animals (Supplementary Fig. 2a), the levels of the CCC components CCDC22 and CCDC93 were markedly reduced in liver homogenates from a COMMD1-deficient (*COMMD1*^*−/−*^) dog (Fig. 1a). Importantly, similar to patients with *CCDC22* mutations, *COMMD1*^*−/−*^ dogs have elevated plasma TC levels, showing a ∼50% increase in plasma cholesterol levels (Fig. 1b) without affecting plasma triglyceride (TG) concentrations (Fig. 1c). In unaffected littermates (*COMMD1*^*+/−*^ dogs), cholesterol is predominantly carried in high-density lipoprotein (HDL), due to the absence of cholesteryl ester transfer protein activity in dogs^20^. In *COMMD1*^*−/−*^ dogs, the plasma cholesterol profile revealed that cholesterol is mainly present in the very low-density lipoprotein (VLDL) and LDL fractions (Fig. 1d).

High levels of copper in the liver have previously been described to be associated with reduced circulating cholesterol levels in mice^21^, we therefore evaluated a possible correlation between progressive hepatic copper accumulation and plasma cholesterol levels in dogs. As a model, we used Labrador retrievers affected by copper toxicosis^22^,^23^ (Supplementary Fig. 2b). The aetiology of this disorder is unknown, but *COMMD1* has been excluded to be the causal gene. Plasma cholesterol levels and lipoprotein profile were unchanged in affected Labrador retrievers (Supplementary Fig. 2c,d). These observations are in agreement with a recent study on Wilson disease, the main disorder of copper toxicosis in humans^24^. Although Wilson disease patients accumulate copper to toxic levels in their livers, as COMMD1-deficient dogs, no unusual plasma cholesterol (total, HDL and LDL) levels were observed^24^. Altogether, these data suggest that aberrant copper homeostasis and elevated plasma cholesterol are not causally linked in dogs or humans, and thus the CCC-deficient phenotype is due to pleiotropic effects.

### Ablation of hepatic *Commd1* increases plasma LDL in mice

**Figure 2:**
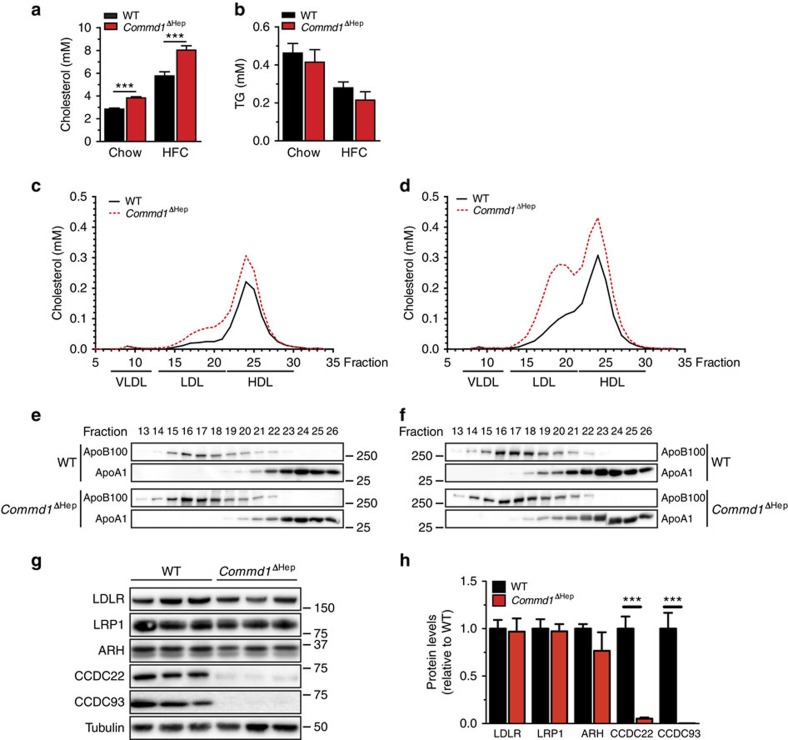
Ablation of hepatic *Commd1* increases plasma total and LDL cholesterol levels. (**a**) Plasma TC and (**b**) TG levels of hepatic *Commd1* knockout mice (*Commd1*^ΔHep^) and WT mice (*n*=6–8) fed a chow diet or a HFC (0.2%) diet for 20 weeks. Pooled plasma samples of each experimental group of mice fed either the (**c**) chow or (**d**) the HFC diet were separated using FPLC gel filtration. Fifty fractions were collected from each separation. TC content was determined and lipoprotein profile was plotted. Fractions #13–26 containing cholesterol were collected and loaded on an SDS polyacrylamide gel and blotted using antibodies against apoA1 and apoB100 lipoproteins. The chow group (**e**) and HFC group (**f**) are shown. (**g**) Livers of chow-fed mice were homogenized, and 30 μg of protein was subjected to immunoblot analysis. Levels of LDLR, LRP1, ARH, CCDC22, CCDC93 and tubulin were determined. Three representative samples from WT and *Commd1*^ΔHep^ mice are shown. (**h**) Relative levels of the proteins shown in **g** were determined by immunoblot analysis, and densitometry analysis was performed using tubulin as a loading control (*n*=6–8 per genotype). The results are presented as mean±s.e.m., and significance was calculated relative to the control group by unpaired Student's *t*-test; ***P<0.001. ARH, autosomal recessive hypercholesterolaemia protein.

LDL is the foremost cholesterol type that is elevated in patients and dogs carrying mutations affecting the CCC complex, and since the liver is the main organ involved in clearing circulating LDL cholesterol^25^ we determined the cholesterol levels and the lipoprotein profiles of hepatocyte-specific *Commd1* knockout mice (*Commd1*^ΔHep^) (ref. 26) fed either a chow diet or a high-fat high-cholesterol (HFC, cholesterol 0.2%) diet. Consistent with the increased plasma cholesterol concentrations found in COMMD1-deficient dogs, plasma cholesterol levels in *Commd1*^ΔHep^ mice were significantly higher than in *Commd1*^loxP/loxP^ wild-type (WT) littermates (Fig. 2a), with an increase of ∼35% (chow-fed mice) and ∼39% (HFC-fed mice). Plasma TG levels, hepatic cholesterol, hepatic TG concentrations and body weight were however unaffected by the loss of hepatic *Commd1* (Fig. 2b and Supplementary Tables 1 and 2). As shown previously^26^, hepatic copper concentrations were not affected in *Commd1*^ΔHep^ mice when dietary copper was not increased (Supplementary Tables 1 and 2), which further supports that the increase in plasma cholesterol by CCC insufficiency is not caused by aberrant copper homeostasis. These data are in agreement with the fact that patients with *CCDC22* mutations have increased serum ceruplasmin and copper levels, but do not exhibit clinical signs of copper overload or liver injury^8^. In addition, we observed no differences in cholesterol levels between WT mice and transgenic mice expressing Cre recombinase in hepatocytes (Alb-Cre), thus excluding the possibility that Cre recombinase expression in murine liver affects cholesterol metabolism (Supplementary Fig. 3a).

Next, we determined the lipoprotein profiles of pooled plasma samples from WT and *Commd1*^ΔHep^ mice fed either conventional chow (Fig. 2c) or HFC diet (Fig. 2d). Independent of the diet, hepatic *Commd1* ablation resulted in significantly higher LDL cholesterol levels (Fig. 2c–f and Supplementary Fig. 3b,c,d,e). To assess whether the increase in plasma LDL in hepatocyte COMMD1-deficient mice is caused by aberrant VLDL homeostasis, VLDL production and secretion were measured. As shown in Supplementary Fig. 4a,b, no differences were found in VLDL production and secretion, indicating that COMMD1 is not involved in VLDL homeostasis. Furthermore, we observed no alterations in hepatic expression of genes and proteins involved in cholesterol uptake, synthesis or efflux (Fig. 2g,h and Supplementary Fig. 4c). However, consistent with results from COMMD1-deficient dogs (Fig. 1a), CCDC22 and CCDC93 levels were both markedly decreased in *Commd1*-ablated hepatocytes (Fig. 2g,h). With the notion that all members of the COMMD family can associate with the CCC complex^8^,^13^,^14^, we assessed if COMMD9 is also involved in cholesterol homeostasis. We therefore deleted *Commd9* specifically in hepatocytes by intravenous injection of recombinant adenovirus carrying Cre recombinase (Ad-Cre) in conditional *Commd9* knockout mice (*Commd9*^loxP/loxP^) (ref. 14). As a negative control mice were injected with adenovirus carrying the *LacZ* gene (Ad-LacZ). Cre-mediated ablation of *Commd9* resulted in a clear reduction in hepatic *Commd9* mRNA and COMMD9 protein levels (Supplementary Fig. 5a,b). Similarly, as shown in hepatocytes lacking COMMD1 (Fig. 2g,h), hepatic COMMD9 inactivation resulted in a decrease in CCDC22 and CCDC93 protein levels (Supplementary Fig. 5b), which was accompanied by an increase in plasma cholesterol levels (Supplementary Fig. 5c). Together, these data indicate that mutations affecting the formation of the CCC complex cause hypercholesterolaemia.

### COMMD1 binds to LDLR through its COMM domain

**Figure 3:**
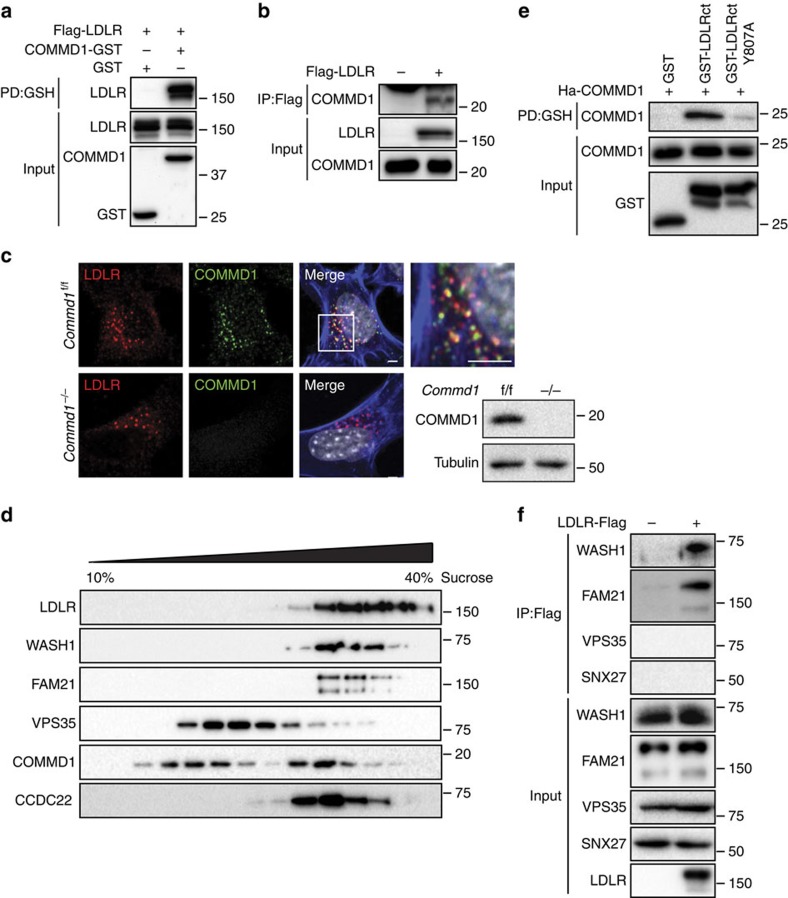
LDLR associates with COMMD1 and the WASH complex. (**a**) Human embryonic kidney 293T (HEK293T) cells were transfected with constructs expressing Flag-LDLR with either COMMD1-GST or GST alone. Interaction with COMMD1 was detected via pull-down assay using glutathione sepharose beads. (**b**) HEK293T cells were transfected with Flag-LDLR vector, and interaction with endogenous COMMD1 was detected by immunoprecipitation with rabbit anti-Flag-antibody. (**c**) Colocalization of LDLR (red) and COMMD1 (green) in *Commd1*^f/f^ MEFs examined by immunofluorescence staining. Representative images are shown; scale bar, 5μm. LDLR (red) and COMMD1 (green) was stained in COMMD1-deficient MEFs (*Commd1*^*−/−*^) and imaged by confocal fluorescence microscopy. COMMD1 levels in *Commd1*^f/f^ and in *Commd1*^*−/−*^ MEFs determined by immunoblot analysis. (**d**) Liver of a WT chow-fed mouse was homogenized and loaded on a continuous 10–40% sucrose gradient. Fractions were separated by ultracentrifugation and immunoblotted using antibodies against COMMD1, LDLR, WASH1, FAM21, VPS35 and CCDC22. The figure represents results of three independent experiments. (**e**) HEK293T cells were transfected with Ha-COMMD1 construct together with GST alone, GST-LDLRct (GST-tagged cytosolic domain of LDLR) or GST-LDLRct Y807A (GST-tagged mutated cytosolic domain of LDLR). Pull-down assay was performed to study the interaction between LDLRct and COMMD1. (**f**) Lysates of Flag-LDLR-transfected HEK293T cells were used for immunoprecipitation assays. Immunoprecipitates were washed, separated by SDS–polyacrylamide gel electrophoresis and immunoblotted as indicated.

Plasma LDL cholesterol levels are largely dependent on functional LDL receptors in the liver, and since the CCC complex mediates the trafficking of various transmembrane proteins^12^,^14^, we investigated whether COMMD1 interacts with LDLR and is required for normal LDLR function. Pull-down and immunoprecipitation assays demonstrated a clear association between COMMD1 and LDLR (Fig. 3a,b). Immunofluorescence staining in mouse embryonic fibroblasts (MEFs) and continuous sucrose gradient fractionation of fresh liver homogenates showed overlap in the cellular distribution of COMMD1 and LDLR, which support a physical association between COMMD1 and LDLR (Fig. 3c,d and Supplementary Fig. 6a). We found that this COMMD1–LDLR interaction depends on the NPxY-based trafficking motif within the cytoplasmic tail of LDLR and on the COMM domain of COMMD1 (Fig. 3e and Supplementary Fig. 6b). Although additional studies are needed to determine whether the interaction between COMMD1 and LDLR is direct, our results are consistent with previous studies, which demonstrated that the NPxY motif is essential for the binding of various LDLR adaptor proteins involved in the intracellular trafficking of LDLR^27^.

### WASH components colocalize and interact with LDLR

Our prior work demonstrated that the CCC complex acts in conjunction with the multi-subunit WASH complex to mediate the trafficking of ATP7A (ref. 8), and this motivated us to assess the cellular distribution of the WASH complex and retromer relative to LDLR. Isopycnic centrifugation of murine liver lysates showed a marked overlap in the cellular distribution of LDLR with WASH components (WASH1 and FAM21), as well as with CCC components (CCDC22 and COMMD1), and to a lesser extent with the retromer component VPS35 (Fig. 3d). Furthermore, immunoprecipitation assays revealed that WASH1 and FAM21 can both bind to LDLR, but no physical association was detected between VPS35 and LDLR (Fig. 3f). In addition, the interaction between the PDZ-domain-containing sortin nexin 27 (SNX27) and LDLR was assessed as SNX27 is required for retrieval and recycling of numerous transmembrane proteins, including several members of the LDLR family (for example, VLDLR)^28^. However, no interaction of LDLR and SXN27 was observed (Fig. 3f). Furthermore, the subcellular localization of SNX27 and its binding to FAM21 was not affected by COMMD1 deficiency (Supplementary Fig. 7). These observations suggest that the COMMD1-containing CCC complex acts in concert with the WASH complex to regulate LDLR endosomal trafficking.

### COMMD1 and WASH facilitate LDLR trafficking

**Figure 4:**
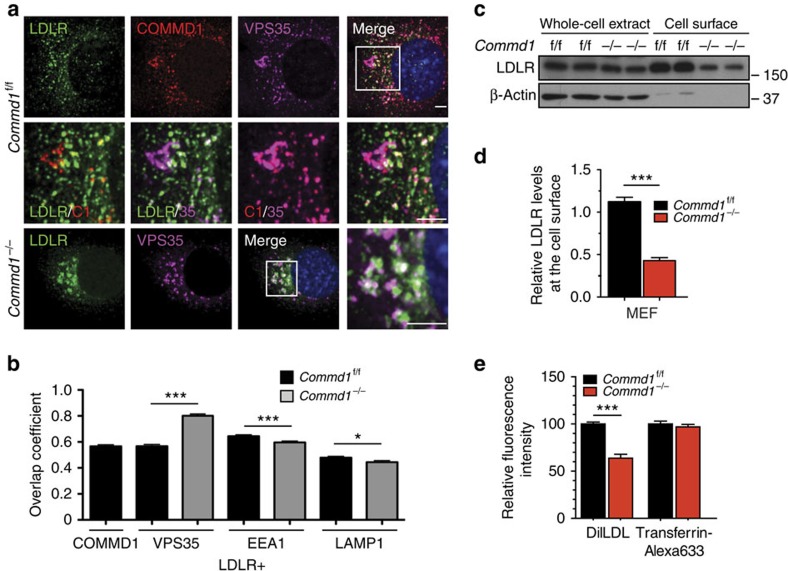
COMMD1 deficiency impairs the function of LDLR. (**a**) LDLR (green), COMMD1 (red) and VPS35 (pink) were stained in *Commd1*^f/f^ and *Commd1*^*−/−*^ MEFs and imaged by confocal microscopy. Representative images are shown; scale bar, 5μm. (**b**) Quantification of the colocalization of LDLR with COMMD1, VPS35, EEA1 and LAMP1 was performed by the analysis of 30–40 cells. (**c**) Total and plasma membrane LDLR levels of *Commd1*^f/f^ and *Commd1*^*−/−*^ MEFs determined by biotinylation assay. Data represent three independent experiments, and (**d**) the relative levels of LDLR at the cell surface are quantified in all experiments. (**e**) *In vitro* LDL and transferrin uptake assay. Dil-labelled LDL (5 μg ml^−1^) or Alexa-633-labelled transferrin (5 μg ml^−1^) was added to serum-depleted medium and incubated with MEFs at 4 °C for 1 h and subsequently at 37 °C for 5 min. Dil-labelled LDL and Alexa-633-labelled transferrin uptake was measured by FACS analysis, and the relative uptake in triplicate is shown. The results are presented as mean±s.e.m.; significance was calculated relative to the control group by unpaired Student's *t*-test; *P<0.05, ***P<0.001.

Next, we examined whether COMMD1 deficiency alters the subcellular localization of LDLR. As observed in HeLa cells^8^, immunofluorescence staining of COMMD1, WASH1 and VPS35 demonstrated marked overlap in MEFs (Supplementary Fig. 8). In normal MEFs, LDLR is present in early endosomes, in EAA1^+^ and VPS35^+^ subdomains, as well as, to some extent, in LAMP1^+^ lysosomes. Loss of COMMD1 decreased the colocalization of LDLR with the early endosomal marker EEA1 and the lysosomal marker LAMP1, but increased LDLR localization to a VPS35-enriched endosomal compartment (Fig. 4a,b and Supplementary Fig. 9a), suggesting that LDLR might not be able to traffic normally from retromer-coated endosomes to other compartments. Next, we quantified surface levels of LDLR in WT and *Commd1*^*−/−*^ MEFs by using a surface biotinylation assay. Although total LDLR levels were not affected by the loss of COMMD1, cell surface levels of LDLR were markedly reduced (Fig. 4c,d). Decreased levels of LDLR was not only observed in *Commd1*^*−/−*^ MEFs but also in primary hepatocytes isolated from *Commd1*^ΔHep^ mice (Supplementary Fig. 9b,c). To assess the functional effect of LDLR mislocalization and decreased LDLR surface levels on LDL cellular uptake, we added fluorescently labelled LDL (Dil-LDL) to the medium and determined the level of endocytosed LDL. We observed a ∼40% decrease in LDL uptake in *Commd1*^*−/−*^ MEFs compared with WT cells (Fig. 4e). To evaluate whether COMMD1 deficiency only affects LDL uptake, we also measured the uptake of labelled transferrin by the transferrin receptor (TfnR). Although the intracellular trafficking of TfnR shows many similarities to LDLR trafficking^29^, no differences in transferrin uptake were observed between *Commd1*^*−/−*^ and WT cells (Fig. 4e). Altogether, these data indicate that COMMD1 is essential for maintaining proper levels of LDLR within the plasma membrane to clear LDL cholesterol.

**Figure 5:**
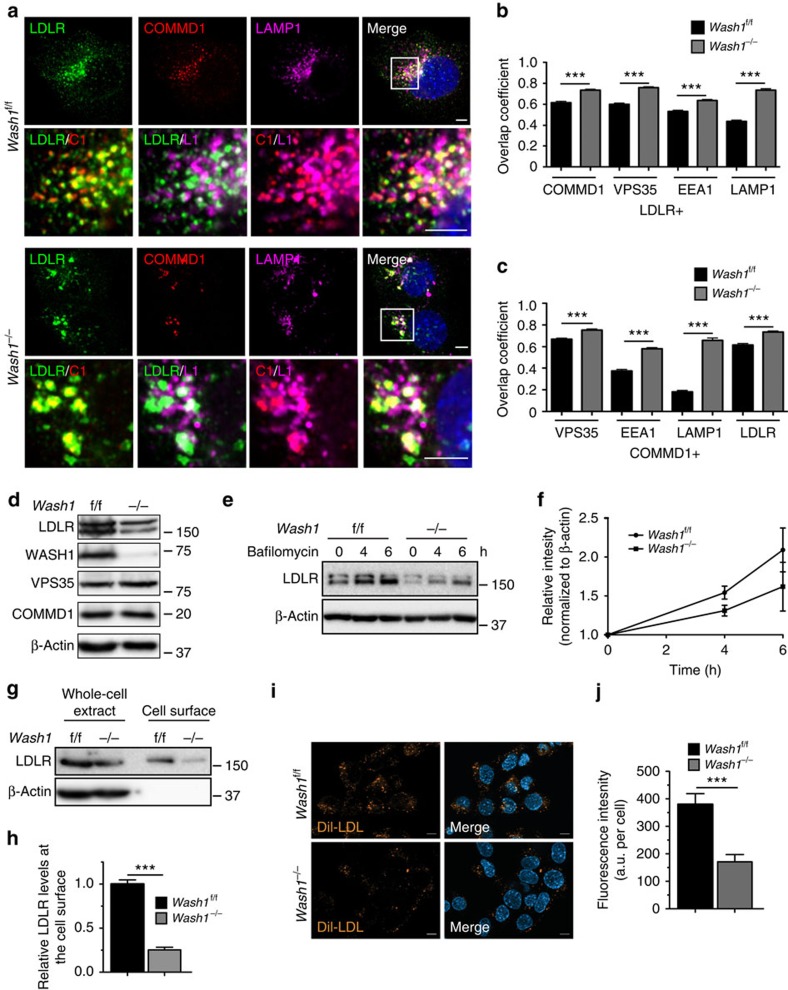
The WASH complex is essential for endosomal sorting of LDLR. (**a**) Cellular localization of LDLR (green), COMMD1 (red) and LAMP1 (pink) in *Wash1*^f/f^ and *Wash1*^*−/−*^ MEFs was determined by immunofluorescence staining. Representative images are shown; scale bar, 5 μm. Relative colocalization of (**b**) LDLR and (**c**) COMMD1 with VPS35, EEA1 and LAMP1 was quantified. (**d**) LDLR, WASH1, VPS35 and COMMD1 levels in *Wash1*^f/f^ and *Wash1*^*−/−*^ MEFs analysed by western blot. (**e**) Representative images (*n*=3) of immunoblot analysis of total LDLR levels in *Wash1*^f/f^ and *Wash1*^*−/−*^ MEFs treated with bafilomycin A (100 nM) for 0, 4 and 6 h. (**f**) Densitometry revealed the relative levels of LDLR in bafilomycin A-treated cells (*n*=3). (**g**) Representative images (*n*=3) of LDLR on the surface of *Wash1*^f/f^ and *Wash1*^*−/−*^ MEFs determined by surface biotinylation assay. (**h**) Densitometry revealed relative LDLR surface levels (*n*=3). (**i**) *Wash1*^f/f^ and *Wash1*^*−/−*^ MEFs were incubated with DiI-LDL for 30 min and imaged by fluorescence microscope. (**j**) Fluorescence intensity was quantified using ImageJ software and was normalized to the number of DAPI nuclei per image; >30 cells per condition were recorded. The results are presented as mean±s.e.m.; significance was calculated relative to the control group by unpaired Student's *t*-test; ***P<0.001.

Since the COMMD1-containing CCC complex is recruited to the endosomes by the WASH complex^8^, we assessed the contribution of the WASH complex on LDLR trafficking by determining the subcellular localization of LDLR in WASH1-deficient (*Wash1*^*−/−*^) MEFs. *Wash1* ablation destabilizes the entire WASH complex^30^, and in WASH-deficient MEFs the colocalization of LDLR and COMMD1 with VPS35, EEA1 and LAMP1 was increased (Fig. 5a–c and Supplementary Fig. 10). In addition, removal of WASH resulted in collapsed endosomal structures, as shown previously^30^. Although COMMD1 levels were not affected by WASH deficiency, LDLR protein levels were markedly reduced (Fig. 5d), but were restored by the treatment with the lysosome inhibitor bafilomycin A (Fig. 5e,f), as shown for epidermal growth factor receptor levels in WASH-deficient cells^30^. In addition to a reduction in total LDLR levels (Fig. 5d), LDLR levels at the cell surface were decreased in *Wash1*^*−/−*^ MEFs, as demonstrated by a surface biotinylation assay (Fig. 5g,h). To assess whether mislocalization and reduced levels of LDLR in *Wash1*^*−/−*^ cells affect LDL uptake, Dil-LDL was added to the media. Dil-LDL uptake was visualized by microscopy and subsequently quantified. WASH1 depletion markedly diminished LDL cellular uptake (Fig. 5i,j). It has been demonstrated that WASH-mediated actin polymerization on the endosomal membrane is crucial for endosomal sorting of specific cargos, and loss of WASH1 results in endosomal collapse (Fig. 5a) (ref. 30). Indeed, restoring WT WASH1 in WASH1-deficient cells rescued the accumulation of LDLR in collapsed endosomes, which could not be prevented by expressing WASH1-ΔVCA, a deletion mutant that cannot activate the Arp2/3 complex (Supplementary Fig. 11). Altogether, these results indicate that WASH-mediated F-actin polymerization on endosomes is essential for the endosomal trafficking of LDLR.

A homozygous splice site mutation in *KIAA0196*, which encodes the WASH component strumpellin, causes Ritscher–Schinzel/3C syndrome (RSS) in a Canadian cohort^31^. This mutation results in a 60% reduction in strumpellin levels^31^, and RSS patients share many clinical features with patients with *CCDC22* mutations, described thus far^18^,^31^. We assessed the plasma cholesterol levels of one molecularly confirmed RSS patient (age 25), and found that the *KIAA0196* mutation was also associated with elevated plasma TC and LDL cholesterol levels exceeding the 95th percentile corrected for age and gender (TC=6.5 mmol l^−1^, HDL=1.5 mmol l^−1^, LDL=4.4 mmol l^−1^ and TG=1.2 mmol l^−1^), comparable to the cholesterol increase seen in patients with *CCDC22* mutations (Table 1). These data indicate that both the CCC and WASH complexes regulate homeostasis of circulating cholesterol through intracellular trafficking of LDLR.

## Discussion

**Figure 6:**
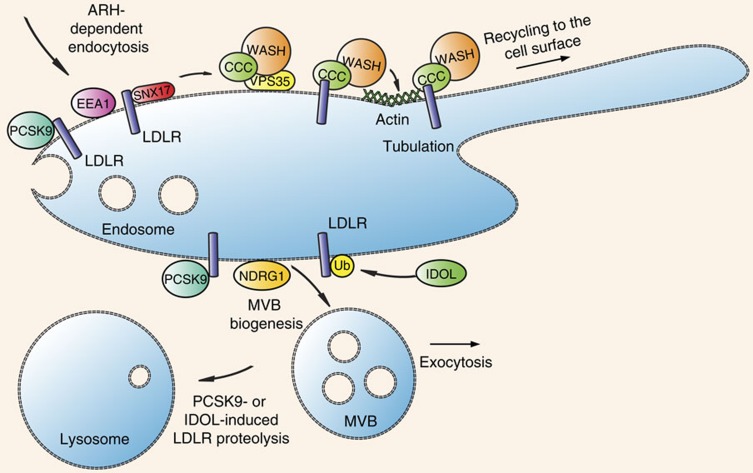
Hypothetical model of CCC- and WASH-mediated endosomal LDLR sorting. After internalization mediated by ARH, LDLR is sorted in endosomes^58^. LDLR is directed to the lysosome for proteolysis—which is induced by either PCSK9 (refs 59, 60) or IDOL^61^—or retrieved and recycled back to the cell surface. SNX17 enhances LDL endocytosis likely by promoting LDLR retrieval^44^, which might precede CCC and retromer/WASH-mediated endosomal trafficking. Through the interaction of the retromer component VPS35 with FAM21 the WASH complex and the CCC complex are recruited to the endosomes^2^,^4^,^8^. Subsequently, CCC and WASH form a protein complex with LDLR; WASH facilitates the formation of branched actin patches to define restricted domains of the endosomes^62^ from where LDLR is sorted back to the cell surface. NDRG1 is required for the formation of MVB: loss of NDRG1 impairs LDL uptake and LDLR recycling, and causes the accumulation of IDOL-mediated ubiquitinated LDLR in MVB^48^. Further research is needed to assess whether CCC and WASH are functionally related to SNX17, NDRG1 and to the lysosomal degradation pathways of LDLR. ARH, autosomal recessive hypercholesterolaemia protein; MVB, multivesicular body; PCSK9, proprotein convertase subtilisin/kexin type 9; Ub, ubiquitin; WASH, WASH1, FAM21, strumpellin, KIAA1033, CCDC53.

The LDL–LDLR axis is a potential therapeutic target for lowering atherogenic plasma cholesterol^32^, and the intracellular route of LDLR has been well described^33^. However, the mechanisms regulating the trafficking of LDLR are not completely understood^34^. Here we identified that the multi-subunit protein complexes CCC and WASH are required for LDLR endosomal sorting and for directing the receptor to the plasma membrane (Fig. 6). Mutations affecting the formation of these complexes are causally related to hypercholesterolaemia in humans, dogs and mice.

COMMD1 was initially identified as a regulator of copper homeostasis in mammals^11^,^26^; however, here we uncover that COMMD1 is also critically important in cholesterol homeostasis. According to our previous work revealing that COMMD1 and the CCC complex regulate the endosomal sorting of the copper transporter ATP7A (ref. 8), we found that this same system similarly regulates the endosomal trafficking of LDLR. We demonstrated this in a variety of cellular models, and the physiologic importance of our observations has been substantiated in two independent mammalian models of COMMD1 deficiency, which display increased plasma LDL cholesterol. In agreement with these models, deleterious human mutations in *CCDC22* (refs 8, 13, 18) are causally linked to elevated plasma total and LDL cholesterol levels in affected patients. The atherogenic lipoprotein phenotype of these patients is similar to that of patients with familial hypercholesterolaemia caused by heterozygous *LDLR* mutations^35^, but cholesterol levels do not reach the high levels seen in patients with homozygous mutations in either *LDLR*, *ARH* (autosomal recessive hypercholesterolaemia) or *APOB* (apolipoprotein B), or in patients with a gain-of-function mutation in *PCSK9* (proprotein convertase subtilisin/kexin type 9) (ref. 35). In addition, elevated TC and LDL cholesterol levels were found in a RSS patient, with a homozygous mutation in the *KIAA0196* gene^31^—encoding for a component of the WASH complex—thus indicating that WASH dysfunction might also cause aberrant cholesterol homeostasis. Therefore, mutations in the *VPS35* gene associated with Parkinson's disease^36^,^37^, and in components of the WASH complex, which are linked to autosomal recessive intellectual disability, and spastic paraplegia^38^,^39^,^40^, warrant future attention with regards to their effects on cholesterol homeostasis.

In endosomal cargo sorting WASH mediates the F-actin formation on endosomes (Fig. 6) (ref. 3), whereas the CCC complex is likely involved in cargo recognition, in which the composition of the CCC complex defines which cargo is specifically sorted. This line of thought is reinforced by the observation that all 10 members of the COMMD family can engage in the CCC complex^13^,^41^, and by our recent work showing that the trafficking of Notch, another cargo of this system, is particularly dependent on COMMD9 in various cell lines^14^. Although these observations advocate for the presence of CCC subcomplexes in cargo selectively trafficking, here we found that deletion of *Commd9* in hepatocytes also causes increased plasma cholesterol in mice, which indicates that the composition of these subcomplexes and their roles in cargo trafficking are likely cell type specific. Further research is therefore warranted to dissect the contribution of the COMMD proteins in cargo selectively trafficking.

The retromer–WASH-associated SNX27 (ref. 42) interacts with the NPxY-based trafficking motif^43^, and here we assessed whether SNX27 is engaged in CCC/WASH-mediated LDLR trafficking. In agreement with prior work^28^, we did not find SNX27 in the interactome of LDLR. Furthermore, ablation of COMMD1 did not affect SNX27–FAM21 interaction or subcellular localization of SNX27. Our results and the findings of Steinberg *et al*.^28^ showing that the depletion of SNX27 in HeLa cells does not affect the levels of LDLR on the cell surface—as shown for other members of the LDLR family—indicate that it is unlikely that SNX27 is involved in LDLR trafficking. Interestingly, the SNX27 family member SNX17 has been identified as an adaptor of LDLR. Like COMMD1, SNX17 binds to the NPxY motif of LDLR^44^,^45^,^46^,^47^ and promotes LDLR endocytosis^44^. SNX17 and COMMD1 both colocalize to vesicles positive for the early endosome marker EEA1 (refs 12, 44), but in contrast to COMMD1, localization studies did not demonstrate overlap between SNX17 and LDLR^44^. Therefore, we hypothesize that the action of SNX17 on LDLR trafficking precedes CCC- and WASH-mediated endosomal sorting of LDLR (Fig. 6). In addition to SNX17, N-myc downstream regulated gene 1 (NDGR1) also regulates the trafficking of LDLR^48^. Loss of NDRG1 reduces LDLR levels on the cell surface accompanied by impaired LDL uptake. In addition, cells deficient for NDRG1 display accumulation of inducible degrader of the LDLR (IDOL)-mediated ubiquitinated LDLR in multivesicular bodies, whose structure is deformed by NDRG1 deficiency (Fig. 6) (ref. 48). Whether CCC and WASH complexes are functionally connected to the aforementioned proteins or to other regulators of the LDLR recycling^34^ warrants further investigation.

Altogether, this study uncovers CCC and WASH as novel multi-subunit protein complexes in the endosomal trafficking of LDLR, and provides valuable insights in the molecular mechanism of cholesterol homeostasis.

## Methods

### Human studies

All participating patients and their family members consented to the evaluations performed. Their participation in this study was approved by the appropriate institutional review boards (Women's and Children's Health Network, Adelaide, South Australia, Australia and Medical University Graz, Graz, Austria), as previously reported^13^,^31^.

### Animals

All animal studies were approved by the Institutional Animal Care and Use Committee, University of Groningen (Groningen, the Netherlands). Hepatocyte-specific *Commd1* knockout mice (*Commd1*^ΔHep^)^26^ were backcrossed for more than eight generations in a C57BL/6J background. All mice were individually housed males, fed *ad libitum* with either a standard rodent chow diet (RMH-B, AB Diets, the Netherlands) or, starting at 8–9 weeks of age, a HFC diet (45% calories from butter fat) containing 0.2% cholesterol (SAFE Diets), *n*=6–8. HFC feeding lasted for 20 weeks. *Commd9*^loxP/loxP^ mice^14^ were retro-orbitally injected with purified 1 × 10^11^ Ad-Cre or Ad-LacZ virus particles^49^. Mice were killed following a 4-h morning fasting period. Tissues for mRNA and protein expression analysis were snap frozen in liquid nitrogen and stored at −80 °C until further analysis. Blood was drawn by heart puncture, and plasma was isolated by centrifugation at 3,000 r.p.m. for 10 min at 4 °C.

Dog samples were partly originated from earlier studies^19^,^22^. Client owned Labrador retrievers were admitted to the Department of Clinical Sciences of Companion Animals, Faculty of Veterinary Medicine, Utrecht University (Utrecht, the Netherlands). Ultrasound-guided liver biopsies were obtained for diagnostic purposes from dogs under general anaesthesia. Copper concentrations in liver biopsies were measured using instrumental neutron activation analysis. Serum samples that were obtained for diagnostic purposes from Labrador retrievers were used for the current study, after informed consent was obtained from the owners. The procedures were approved by Utrecht University Ethical Committee, as required under Dutch legislation.

### Cholesterol and TG analysis in plasma and liver homogenates

TC levels were determined using colorimetric assay (11489232, Roche Molecular Biochemicals) with cholesterol standard FS (DiaSys Diagnostic Systems) as a reference. TG levels were determined using Trig/GB kit (1187771, Roche Molecular Biochemicals) with Roche Precimat Glycerol standard (16658800) as a reference.

### Hepatic lipid extraction

Liver homogenates prepared as 15% (w/v) solutions in PBS were subjected to lipid extraction according to the Bligh & Dyer method^50^. In short, 600 μl of demi-water was mixed with 200 μl of liver homogenate, and 3 ml of chloroform/methanol was added and mixed. After 30 min of incubation 1.2 ml of H_2_O and 1 ml of chloroform were added, mixed and subsequently centrifuged (10 min, 1,500 r.p.m. at room temperature). The chloroform layer was transferred into a new glass tube, and was evaporated using nitrogen at 50 °C. Lipids were resolved in 1 ml of chloroform and used for further determination of cholesterol and TG content.

### *In vivo* VLDL-TG production

The experiment was performed on 10- to 13-week-old chow-fed mice. After a 4-h morning fasting, animals were intraperitoneally injected with poloxamer 407 (BASF) solution in saline (1 g per kg body weight). Blood was drawn by retro-orbital puncture at the following time points: 0, 30, 60, 120 and 240 min. These samples were used for TG determination and calculation of VLDL-TG production rate.

### Antibodies

In the experimental procedures described, the following antibodies were used: rabbit polyclonal antibody against COMMD1 (11938-1-AP, Proteintech Group, 1:1,000); rabbit polyclonal antibody against LDLR (PAB8804, Abnova, 1:1,000); rabbit polyclonal antibody against GST (Z-5) (sc-459, Santa Cruz Biotechnology, 1:5,000); goat anti-rabbit IgG (H+L)-horseradish peroxidase (HRP) conjugate (170-6515, Bio-Rad Laboratories, 1:10,000); goat anti-mouse IgG (H+L)-HRP conjugate (170-6516, Bio-Rad Laboratories, 1:10,000); mouse anti-β-actin (A5441, Sigma-Aldrich, 1:5,000); rabbit anti-tubulin (AB4047, Abcam, 1:2,000); rabbit anti-Rab11 (700184, Invitrogen, 1:1,000); rabbit anti-EEA-1 (AB2900, Abcam, 1:1,000); rabbit anti-CCDC22 (16636-1-AP, Proteintech, 1:2,000); and rabbit anti-CCDC93 (20861-1-AP, Proteintech, 1:5,000). Rabbit polyclonal antibodies against WASH1, FAM21 and VPS35 were previously described^1^,^5^. Anti-apoB100 (1:1,000) and rabbit anti-apoA1 (1:1,000) antibodies were a gift from A.K. Groen. Rabbit polyclonal antibody against ARH (1:1,000) was a gift from H.H. Hobbs. Densitometry analysis of western blot bands was performed using Image Lab 3.0.1 software (Bio-Rad Laboratories). Images have been cropped for presentation. Full-size images are presented in Supplementary Fig. 12.

### Expression constructs

The following vectors were used in the experiments described: pEBB-COMMD1-Flag; pEBB-GST; pEBB-COMMD1-GST; pEBB-1-118-GST; and pEBB-119-190-GST^10^,^51^. Full-length Flag-tagged LDLR receptor, GST-LDL cytoplasmic tail (-cyt) and GST-LDL-cyt Y807A were obtained from N. Freedman^52^, and Flag-LDLR was subcloned into pDNA3.1. WT and mutant WASH1-GFP constructs have been described before^30^.

### Immunofluorescence staining

Staining was carried out as previously described^1^. Cells were fixed in ice-cold fixative (4% paraformaldehyde and 0.5% glutaraldehyde in PBS) and incubated for 18 min at room temperature in the dark, followed by permeabilization with 0.2% Triton X-100 in PBS for 4 min. Cells were then cultured with primary antibodies in IF buffer (TBS plus human serum cocktail) overnight at 4 °C in a humidifier chamber. Next day, cells were washed three times in PBS, and cells were incubated with secondary antibodies in blocking buffer for 1 h at room temperature. After four washes in PBS and addition of Hoechst 33342 nuclear stain, coverslips were rinsed in water and affixed to slides with Slowfade Antifade reagent (Invitrogen). Images were obtained with an LSM-710 laser scanning confocal microscope with a × 100/1.4 Oil Plan-Aprochromat objective lens using ZEN software (Carl Zeiss). Quantitative Pearson's correlation coefficients were obtained using Zen 2009 software (Carl Zeiss) by drawing a region of interest around the objects. The following antibodies were used: rabbit polyclonal antibody against LDLR (PAB8804, Abnova, 1:200); mouse monoclonal antibody against COMMD1 (MAB7526, R&D Systems, 1:400); rabbit polyclonal antibody against EEA1 (2411, Cell Signaling Technologies, 1:250); goat polyclonal antibody against VPS35 (ab10099-100, Abcam, 1:1,000); rat monoclonal antibody against LAMP1 (553792, BD Biosciences, 1:500); rabbit polyclonal antibody against WASH1 (1:1,000); and rabbit polyclonal antibody against FAM21 (1:1,000) (ref. 1).

### Gene expression analysis

Pieces of murine liver of ∼100 mg were homogenized in 1 ml QIAzol Lysis Reagent (Qiagen, Venlo, the Netherlands). Total RNA was isolated by chloroform extraction. Isopropanol-precipitated and ethanol-washed RNA pellets were dissolved in RNase-/DNase-free water. RNA (1 μg) was used to prepare cDNA with the Quantitect Reverse Transcription Kit (Qiagen), according to the manufacturer's protocol. cDNA (20 ng) was used for subsequent quantitative real-time PCR analysis using iTaq SYBR Green Supermix with ROX (Bio-Rad Laboratories BV) and 7900HT Fast Real-Time PCR Systems (Applied Biosystems). The following programme was used: 50 °C/2 min; 95 °C/10 min; 40 cycles with 95 °C/15 s; and 60 °C/1 min. Expression data were analysed using SDS 2.3 software (Applied Biosystems) and the standard curve method of calculation. Mouse cyclophilin A was used as an internal control gene. Primers used for the expression studies are listed in Supplementary Table 3.

### Fast-performance liquid chromatography

Plasma samples within each murine or canine experimental group were pooled together and fractionated using the fast-performance liquid chromatography (FPLC) method. All 50 fractions were analysed to determine TC and TG content. Fractions containing LDL and HDL were further analysed by immunoblot using anti-apoA1 (1:1,000) and anti-apoB100 antibodies (1:1,000).

The TC distribution among the main lipoprotein classes of the individual plasma samples was measured using FPLC analysis as described previously but with some minor modifications^53^. In brief, the system contained a PU-980 ternary pump with an LG-980-02 linear degasser and an UV-975 UV/VIS detector (Jasco). An extra PU 2080i-plus pump (Jasco) was used for in-line cholesterol ready-to-use enzymatic reagent (Biomerieux) addition at a flow rate of 0.1 ml min^−1^. EDTA plasma was diluted 1:1 with Tris-buffered saline, and 30 μl sample buffer mixture was loaded on a Superose 6 HR 10/30 column (GE Healthcare) for lipoprotein separation at a flow rate of 0.31 ml min^−1^. Chromatographic profiles of commercially available plasma lipid standards (SKZL) served as reference.

### Cell culture

Human embryonic kidney 293T cells (American Type Culture Collection) and MEFs^30^,^54^ were cultured using Dulbecco's Modified Eagles Medium (DMEM) with Glutamax (GIBCO), supplemented with 10% fetal bovine serum (Invitrogen) and antibiotics. Primary mouse hepatocytes were cultured in DMEM—low glucose (GIBCO), supplemented with 5% fetal calf serum (FCS) or normal calf lipoprotein-poor serum (NCLPPS) and antibiotics.

### Sucrose gradients

Sucrose gradient separation of fractions obtained from fresh liver tissue was performed as described before^55^ with few modifications. Chow-fed mice were fasted for 4 h before killing. A unit of 200 mg of liver was homogenated in 800 μl homogenization buffer (50 mM Tris-HCl, ph 7.4, 250 mM sucrose, 25 mM KCl, 5 mM MgCl_2_, 3 mM imidazole and protease inhibitor mixture) using a Douncer homogenizer. Homogenates were centrifuged 1,000*g*, 10 min at 4 °C. A unit of 300 μg liver homogenate was loaded on a 3.7-ml continuous 10–40% sucrose gradient, and were centrifuged for 16 h using a Beckman Coulter ultracentrifuge equipped with a swinging bucket rotor SW55 Ti, at 40,000 r.p.m., and fractions of 285 μl were collected from the top of the tube; 1/10 of each fraction was mixed with SDS sample buffer and used for immunoblot analysis.

### Biotinylation assay

Culture of primary hepatocytes and biotinylation assay were performed as previously described^56^. Cells were washed (3 × ) with ice-cold PBS-CM buffer (PBS, 1 mM MgCl_2_ and 0.1 mM CaCl_2_), subsequently 0.5 mg ml^−1^ biotin reagent solution (EZ-Link Sulfo-NHS-SS_Biotin, Thermo Scientific) in biotinylation buffer (10 mM triethanolamine, pH 8.0, 150 mM NaCl and 2 mM CaCl_2_) was added to the cells for 30 min at 4 °C. Biotin reagent was removed and cells were washed 1 × with quenching buffer (PBS-CM, 25 mM Tris-HCl, pH 7.4 and 192 mM Glycine) for 30 min at 4 °C. Next, cells were washed 2 × with PBS-CM and 1 × with TBS-C (50 mM Tris-Cl, pH 7.4, 100 mM NaCl and 2 mM CaCl_2_), and cells were collected by scraping in TBS-C. Cells were centrifuged (1,000*g*, 5 min, 4 °C), lysed in biotin lysis buffer (50 mM Tris-Cl, pH 7.4, 150 mM NaCl, 1% NP-40, 0.5% sodium deoxycholate, 5 mM EDTA and 5 mM EGTA), sonicated (10% output for 10 s) and incubated on ice for 15 min, subsequently centrigufed for 15 min at 12,000 *g*. Protein concentration was determined; 30 μg was used as input; 300 μg was used diluted in biotin lysis buffer (500 μl); 30 μl Neutravidin beads (Neutravidin plus ultralink beads, Thermo Scientific) were added and incubated for 4 h at 4 °C. Beads were collected by centrifugation (500*g*, 5 min), beads were washed 3 × with Biotin lysis buffer, 1 × with high-salt buffer (50 mM Tris-Cl, pH 7.4 and 500 mM NaCl) and 1 × with low-salt buffer (10 mM Tris-HCl). Finally the beads were resuspended in 30 μl 2 × loading buffer.

### Immunoprecipitation analysis

The glutathione *S*-transferase (GST) pull-down assays and immunoprecipitation experiments were performed as described before^57^. Cells were mainly lysed in NP-40 buffer (0.4 M NaCl, 0.1% NP-40, 10 mM Tris-HCl, pH 8.0 and 1 mM EDTA) supplemented with protease inhibitors. Immunoprecipitation of the WASH complex and retromer was performed in MRB lysis buffer (20 mM HEPES pH 7.2, 50 mM potassium acetate, 1 mM EDTA, 200 mM D-sorbitol and 0.1% Triton X-100) supplemented with protease inhibitors. Equal amount of protein was used for immunoprecipiation and pull-down assays. FLAG M2 agarose beads (Sigma-Aldrich) were used for Flag-immunoprecipitation experiments, Glutathione Sepharose 4B (GE Healthcare Life Sciences) for GST pull-down assays. Flag-, Ha-, GST-tagged proteins were detected by Flag-antibody (F1804, Sigma, 1:2,000), Ha-antibody (clone HA-7, H6533, Sigma-Aldrich, 1:5,000) and GST-antibody (sc-138, Santa Cruz, 1:5,000), respectively.

### Dil-LDL uptake assay

Culture medium was replaced with DMEM culture medium supplemented with Dil-LDL (5 μg ml^−1^; Molecular Probes, Invitrogen). Cells were kept for 1 h at 4 °C, then for 5 min at 37 °C. Then they were immediately placed on ice, washed with cold PBS and scraped. As a control for the specificity of the investigated uptake pathway, cells were incubated with Alexa633-transferrin (5 μg ml^−1^). Cells were centrifuged at 300*g* for 5 min at 4 °C and resuspended in 50 μl of FACS buffer (PBS with 2% FCS and 5 mM EDTA) supplemented with additional 5% fetal bovine serum. Cell pellets were vortexed, and 2 ml of FACS buffer was added; samples were centrifuged as before and resuspended in 200 μl of FACS buffer. Cells were kept on ice at all times and subjected to immediate FACS analysis. The number of positive cells was counted and recorded as a percentage of the whole population. Data of four independent experiments were presented relative to the uptake results of the control WT population (set as 100%). Dil-LDL uptake in MEFs determined by fluorescence microscopy was performed with MEFs cultured for 16 h in DMEM medium supplemented with lipoprotein-deficient serum (10%). Medium was changed with Dil-LDL (5 μg ml^−1^) containing 5% lipoprotein-deficient serum/DMEM medium, and after 30 min cells were fixed and mounted with Vectashield mounting medium with DAPI (Vector Laboratories). Images were acquired with a Zeiss AxioObserver.Z1. Fluorescence intensity was quantified using ImageJ software (National Institutes of Health) and was normalized to the number of DAPI nuclei per image; >30 cells per condition were recorded.

### Statistical analysis

All results are expressed as a mean±s.e.m. Statistical analysis was performed using Prism 5.00 for Windows (GraphPad Software) and the unpaired Student's *t*-test. Results of *P*<0.05 were considered statistically significant: *P<0.05, **P<0.01 and ***P<0.001.

## Additional information

**How to cite this article:** Bartuzi, P. *et al*. CCC- and WASH-mediated endosomal sorting of LDLR is required for normal clearance of circulating LDL. *Nat. Commun.* 7:10961 doi: 10.1038/ncomms10961 (2016).

## Supplementary Material

Supplementary Information Supplementary Figures 1-12, Supplementary Tables 1-3 and Supplementary References

## Figures and Tables

**Table 1:** Plasma lipid levels of individuals with mutations in *CCDC22*.

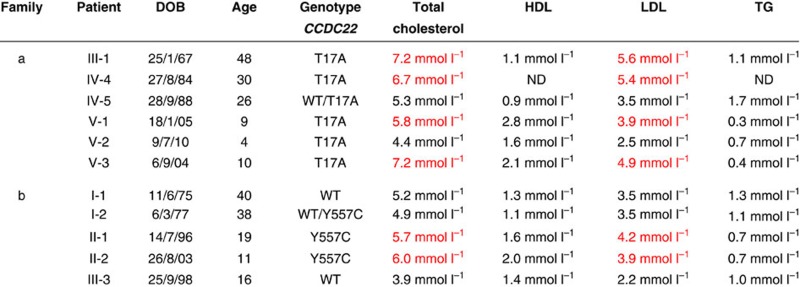

DOB, date of birth; ND, not determined.

In red levels >95th percentiles corrected for age and gender. The age is indicated at the moment of clinical evaluation.
