# Supplementary material for: CCC- and WASH-mediated endosomal sorting of LDLR is required for normal clearance of circulating LDL
Source: Nat Commun. 2016 Mar 11;7:10961. doi: 10.1038/ncomms10961 (PMC4792963; doi:10.1038/ncomms10961)
Supplement: Supplementary Information — Supplementary Figures 1-12, Supplementary Tables 1-3 and Supplementary References [file ncomms10961-s1.pdf]

Supplementary Figure 1

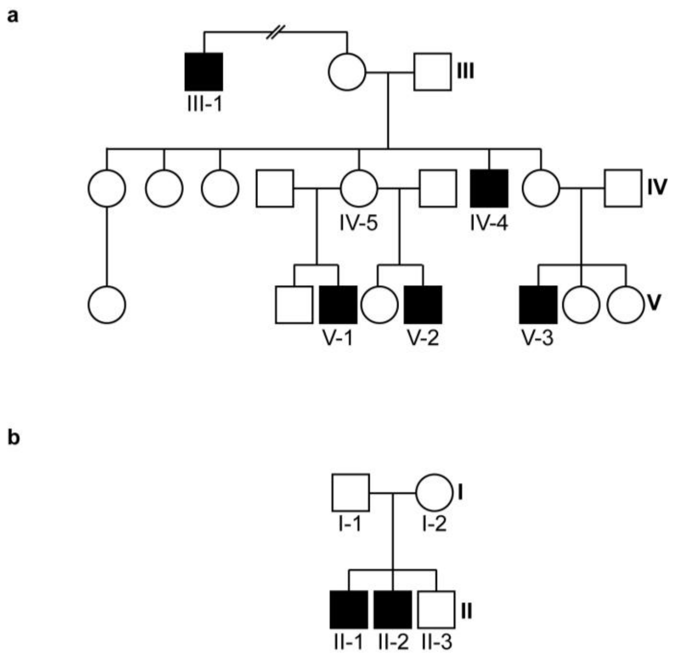

Supplementary figure 1. Pedigrees of affected individuals with *CCDC22* mutations. (**a**) XLID family with T17A mutation. This pedigree is partly adapted from Figure 1E, published by Phillips-Krawczak et al.[1] (**b**) XLID family with Y557C mutation[2].

Supplementary Figure 2.

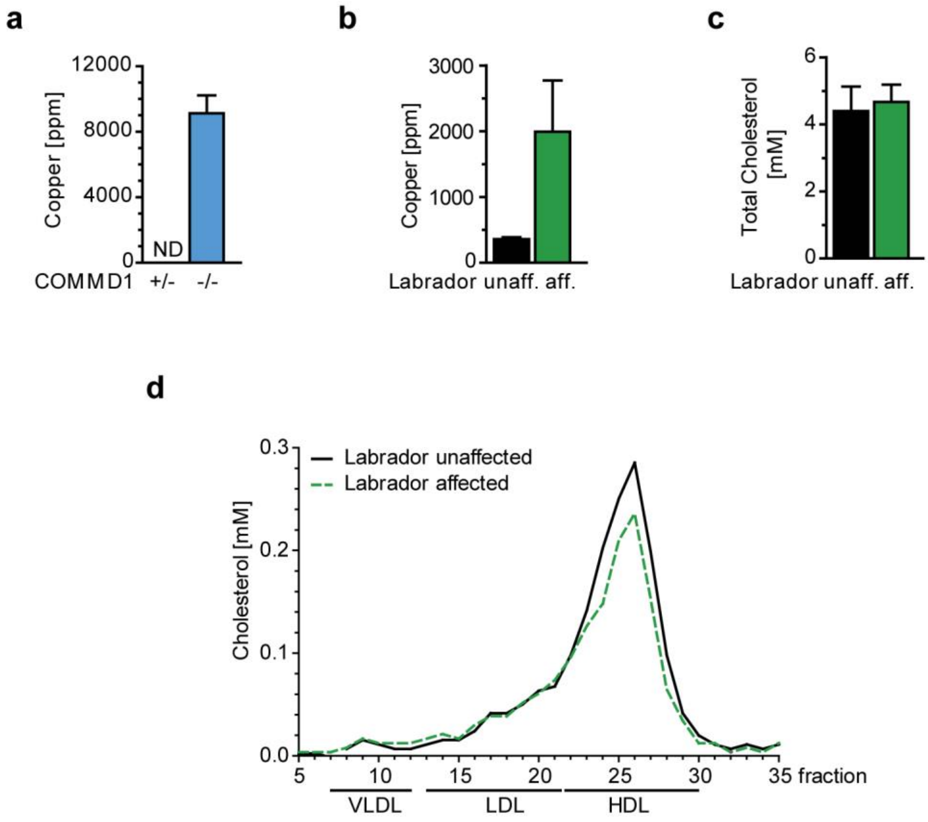

Supplementary figure 2. Elevated hepatic copper levels do not affect cholesterol homeostasis in dogs. (**a**) Hepatic copper levels in carriers (*COMMD1*$^{+/-}$) and *COMMD1*$^{-/-}$ dogs. (**b**) Hepatic copper levels (**c**) and plasma cholesterol concentrations in unaffected (n = 4) and affected Labrador retrievers (n = 5). (**d**) Lipoprotein profiles of unaffected and affected Labrador retrievers.

Supplementary Figure 3

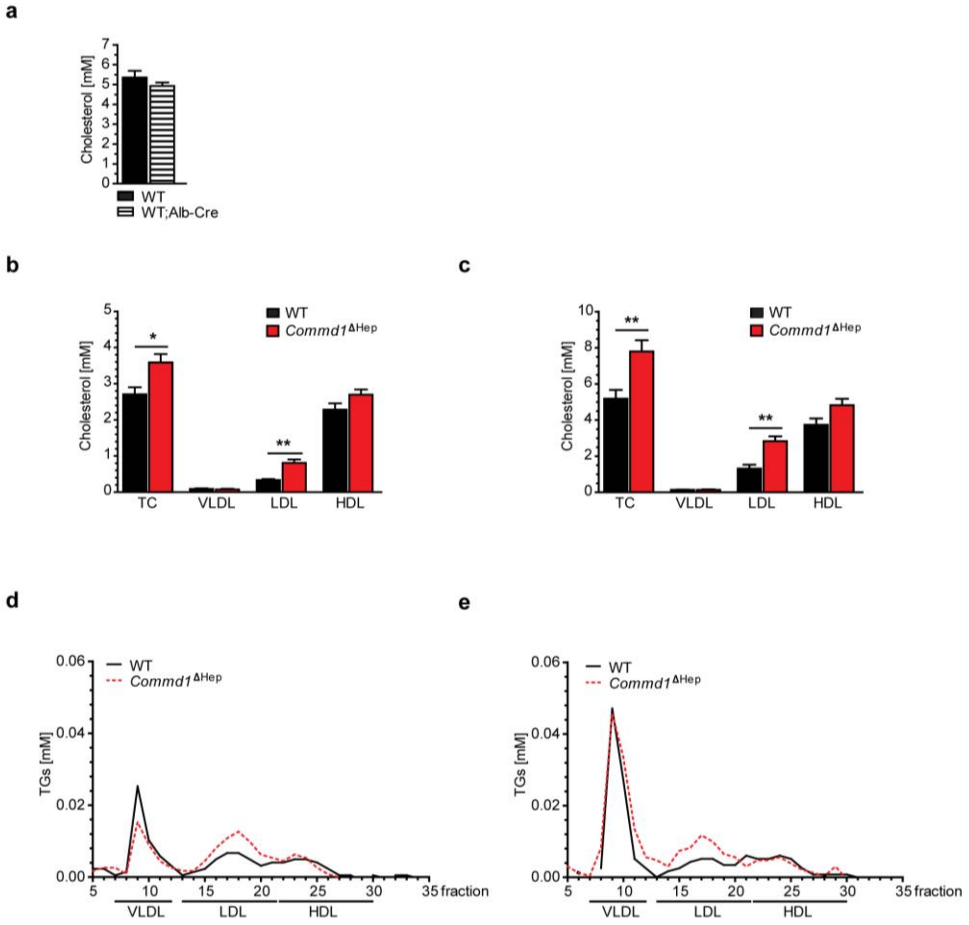

Supplementary figure 3. Plasma cholesterol and lipid levels are affected by hepatic Commd1 deficiency. (**a**) Plasma cholesterol levels of HFC-fed (20 weeks) WT (n = 7) and Alb-cre mice (n=8). Total cholesterol and cholesterol concentration in the different lipoproteins (VLDL, LDL and HDL) of chow-fed (**b**) and HFC-fed (20 weeks) (**c**) WT and *Commd1*$^{\Delta Hep}$ mice (n=6–8). Plasma triglyceride (TG) distribution in the different lipoproteins (VLDL, LDL, HDL) of chow-fed (**d**) and HFC-fed (20 weeks) (**e**) WT and *Commd1*$^{\Delta Hep}$ mice (n=6–8). The results are presented as mean ± s.e.m.; significance was calculated relative to the control group by unpaired Student’s t-test; *P < 0.05, **P < 0.01.

Supplementary Figure 4

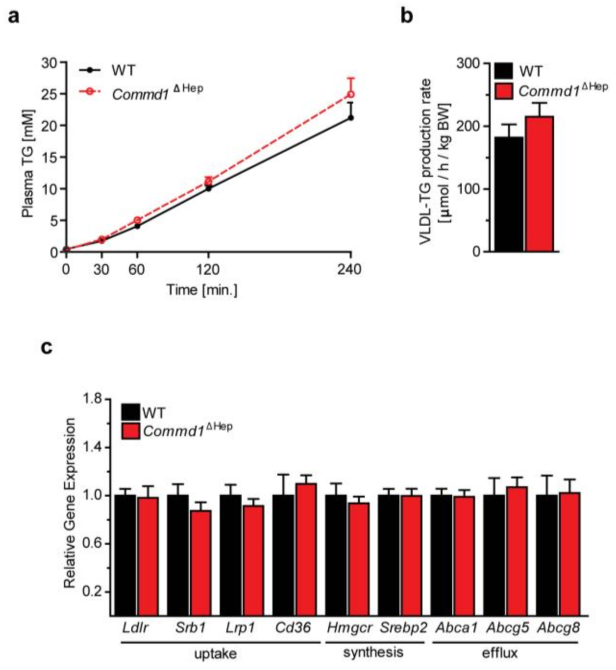

Supplementary figure 4. VLDL-TG production and hepatic expression of LDLR and other lipid-related genes are not affected by the loss of hepatic COMMD1. (**a**) After intraperitoneal injection of poloxamer 407, blood was drawn via orbita puncture at the following time points: 0, 30, 60, 120, and 240 min. TG concentration was determined in plasma from WT *Commd1*$^{\Delta Hep}$ mice (n=6–8) and plotted against time. (**b**) VLDL-TG production rate was calculated based on the TG concentration curve and corrected for the total time of experiment (4 h) and body weight of mice. (**c**) Hepatic mRNA levels of genes involved in cholesterol uptake, synthesis, and efflux were analyzed in chow-fed WT and *Commd1*$^{\Delta Hep}$ mice (n=6–8) and presented relative to the WT control group. Group mean values are presented ± s.e.m.

Supplementary Figure 5

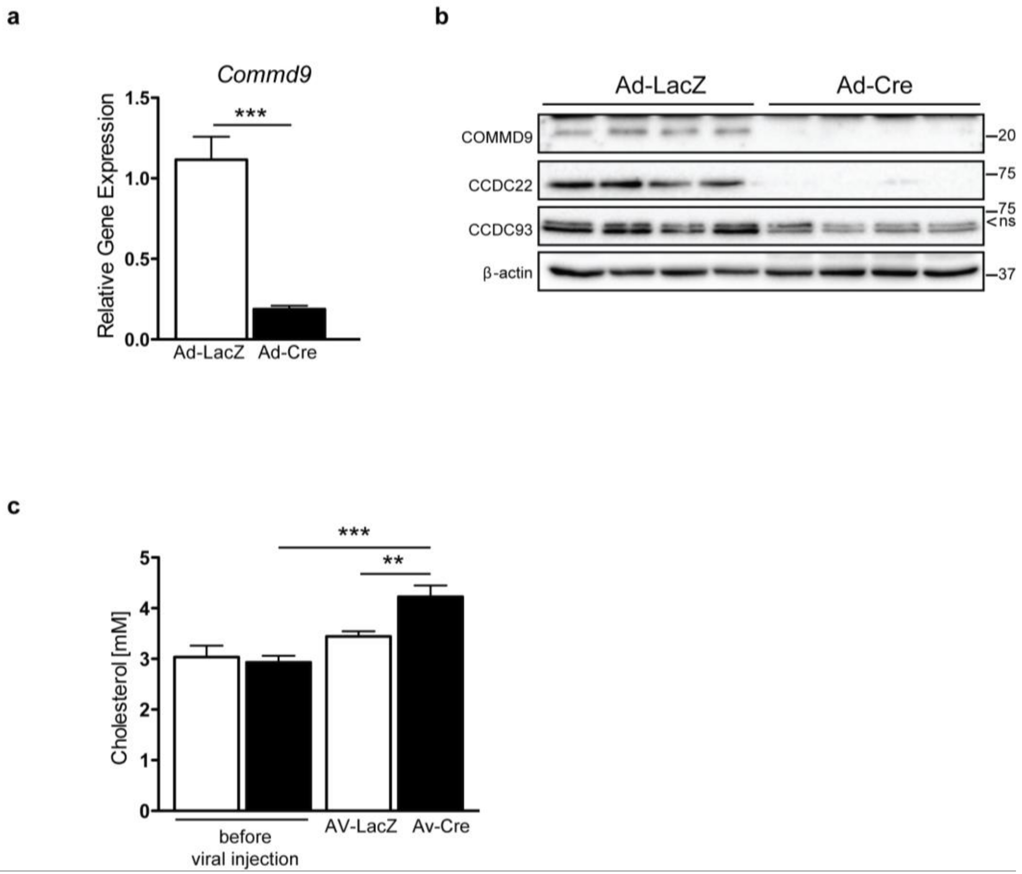

Supplementary figure 5. Hepatocyte Commd9 depletion results in elevated plasma cholesterol levels. (**a**) Relative liver mRNA expression of *Commd9* in *Commd9*$^{loxP/loxP}$ mice injected with control adenovirus (Ad-LacZ) or adenovirus carrying Cre recombinase (Ad-cre) (n=6-7). (**b**) Protein levels of the indicated proteins in livers of *Commd9*$^{loxP/loxP}$ mice injected with either Ad-LacZ or Ad-Cre, determined by immunoblot analysis. Four representative samples from Ad-LacZ or Ad-Cre injected mice are shown (**c**) Total plasma cholesterol levels of mice one week before and 3 weeks after adenoviral injections (n=6-7). The results are presented as mean ± s.e.m.; significance was calculated relative to the control group by unpaired Student's t-test; $**P < 0.01$, $***P < 0.001$.

Supplementary Figure 6.

a

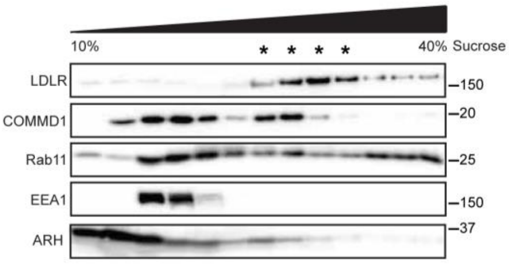

b

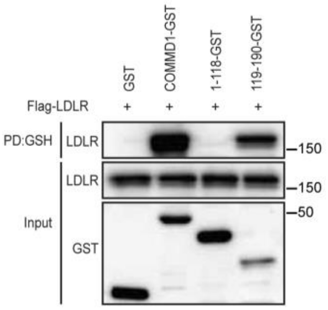

Supplementary figure 6. COMM domain of COMMD1 is essential for the binding to LDLR. (**a**) Relative distribution of COMMD1 in hepatocytes. A liver of a WT chow-fed mouse was homogenized and loaded on a continuous 10–40% sucrose gradient. Fractions were separated by ultracentrifugation and immunoblotted as indicated. EEA1: the late endosomal marker, ARH (autosomal recessive hypocholesterolemia protein): the LDLR adaptor protein ARH, and RAB11: the recycling endosome marker. The star indicates the fractions containing both COMMD1 and LDLR. (**b**) HEK293T cells were transfected with Flag-LDLR vector together with either GST alone, COMDM1-GST, 1–118-GST (GST-tagged COMMD domain) or 119–190-GST (GST-tagged N-terminal region of COMMD1). Interaction with LDLR was detected by pull-down assay using glutathione sepharose beads.

Supplementary Figure 7.

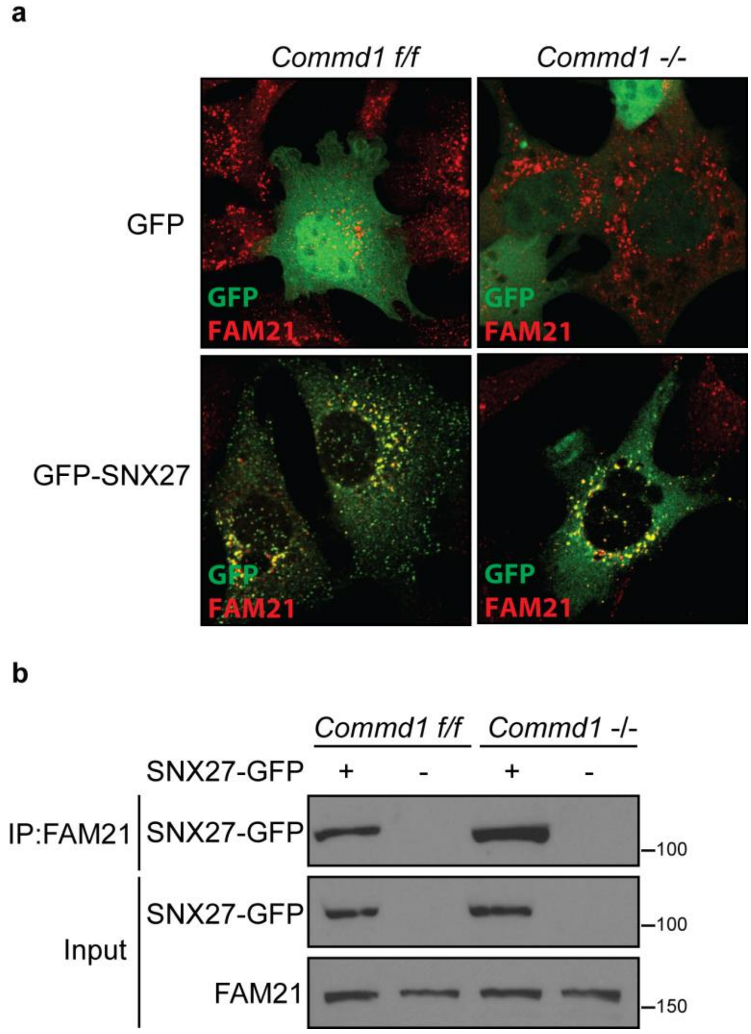

Supplementary figure 7. SNX27 localization and binding to FAM21 are unaffected by COMMD1 ablation. (a) Colocalization of GFP (green) and GFP-SNX27 (green) with FAM21 (red) in wild type (*Commd1*$^{f/f}$) and COMMD1 deficient (*Commd1*$^{-/-}$) MEFs. (**b**). *Commd1*$^{f/f}$ and *Commd1*$^{-/-}$ MEFs were transfected with empty vector or GFP-SNX27 vector and used for immunoprecipitation assay as indicated.

Supplementary Figure 8.

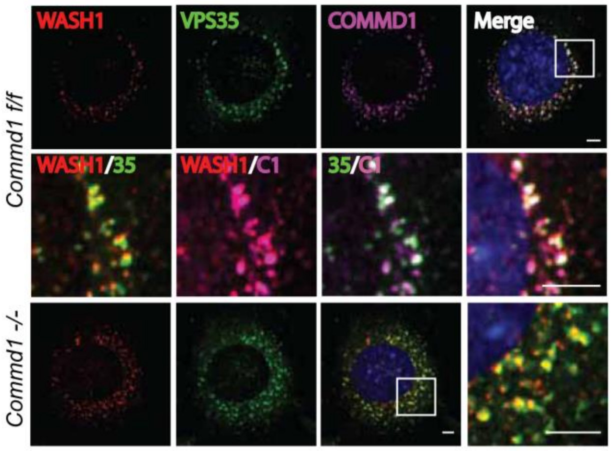

Supplementary figure 8. COMMD1 colocalizes with WASH and the retromer component VPS35. Subcellular localization of WASH1 (red), VPS35 (green), and COMMD1 (pink) in *Commd1*$^{f/f}$ and *Commd1*$^{-/-}$ MEFs by indirect immunofluorescence staining. Representative images are shown; scale bar, 5μm.

Supplementary Figure 9.

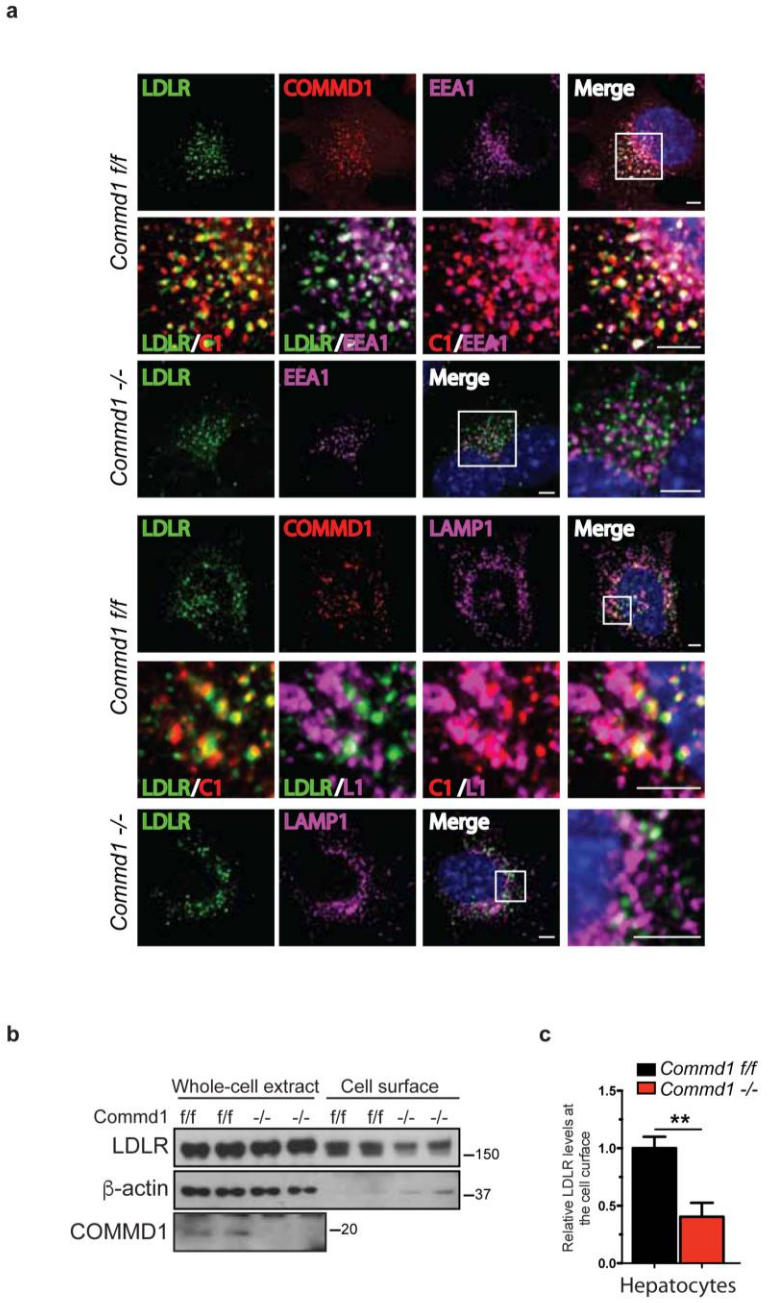

Supplementary figure 9. COMMD1 deficiency affects cellular distribution of LDLR. (**a**) Confocal microscopy was used to determine the subcellular localization of LDLR, EEA1, LAMP1, and COMMD1 (as indicated) in *Commd1*$^{f/f}$ and *Commd1*$^{-/-}$ MEFs. Merged pictures and insets are depicted as indicated. Representative images are shown; scale bar, 5μm. (**b**) Total and plasma membrane LDLR levels of *Commd1*$^{f/f}$ and *Commd1*$^{-/-}$ primary hepatocytes determined by biotinylation assay.

Data represent three independent experiments, and (**c**) relative LDLR surface levels are quantified of three independent experiments. The results are presented as mean ± s.e.m.; significance was calculated relative to the control group by unpaired Student's t-test; $**P < 0.01$.

Supplementary Figure 10.

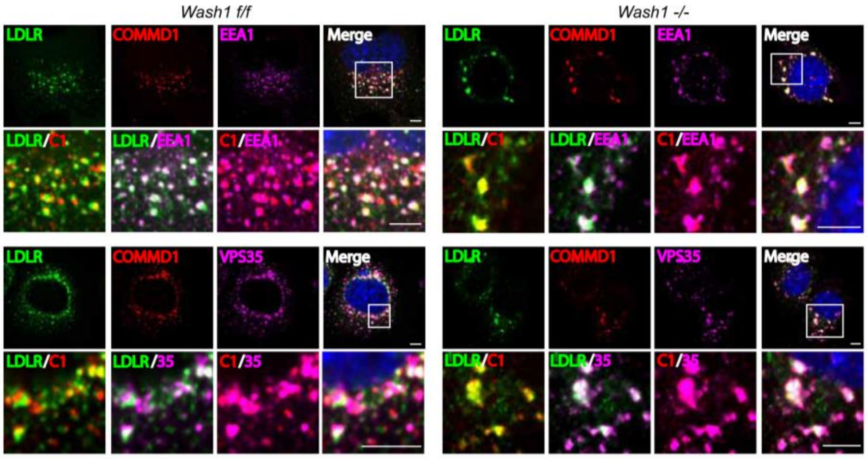

Supplementary figure 10. Subcellular localization of COMMD1 and LDLR are affected by WASH depletion. *Wash1*$^{f/f}$ and *Wash1*$^{-/-}$ MEFs were analyzed by immunofluorescence staining. Merged pictures and insets are displayed as indicated, scale bar, 5μm.

Supplementary Figure 11.

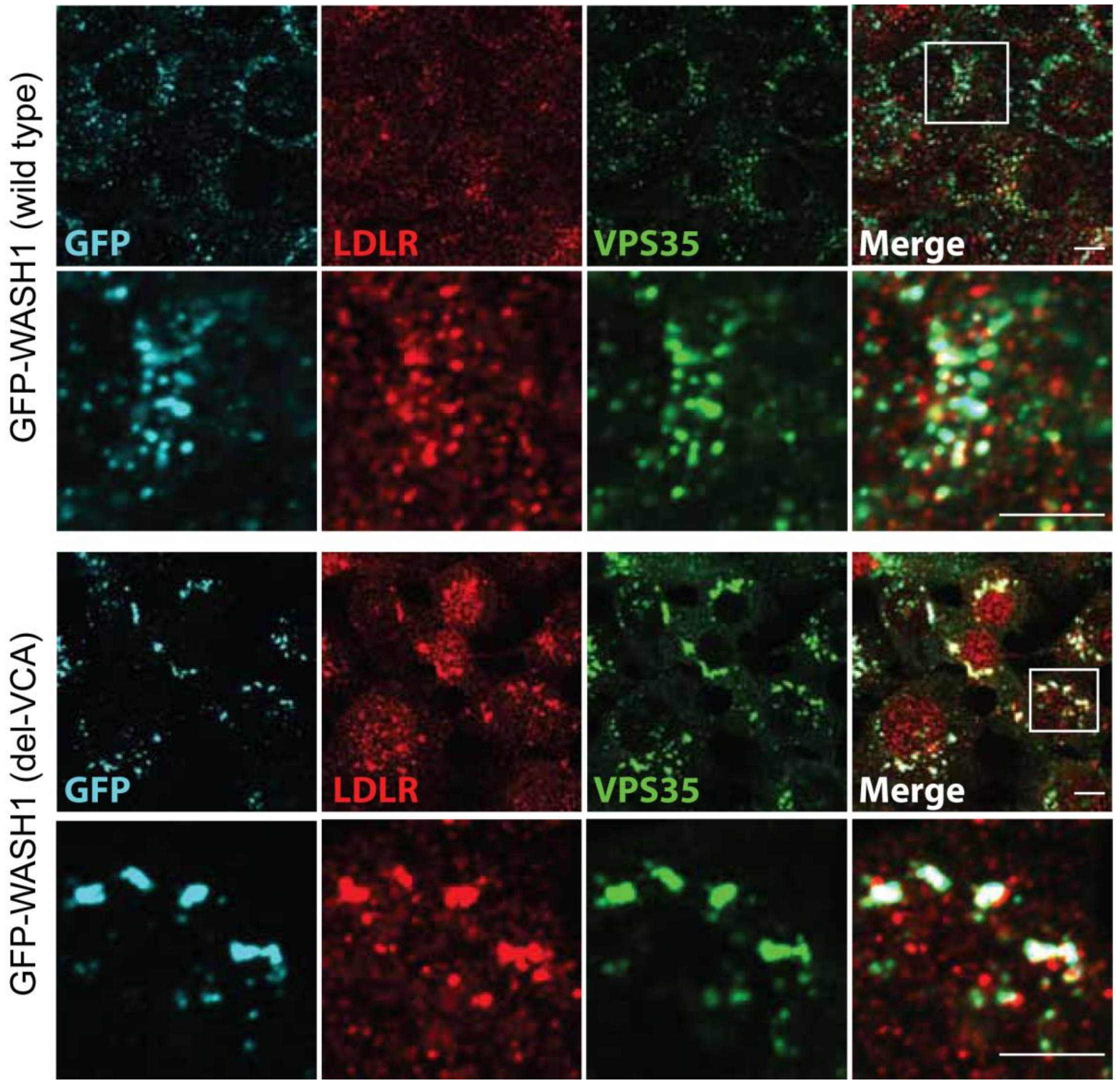

Supplementary figure 11. Endosomal sorting of LDLR depends on WASH-mediated F-actin polymerization. The subcellular localization of LDLR (red), VPS35 (green), and GFP-WASH1 (cyan) were determined by fluorescence microscopy in *Wash1*$^{-/-}$ MEFs reconstituted with GFP-WASH1 or GFP-WASH1 ΔVCA. Representative images are shown; scale bar, 5 μm.

Supplementary Figure 12.

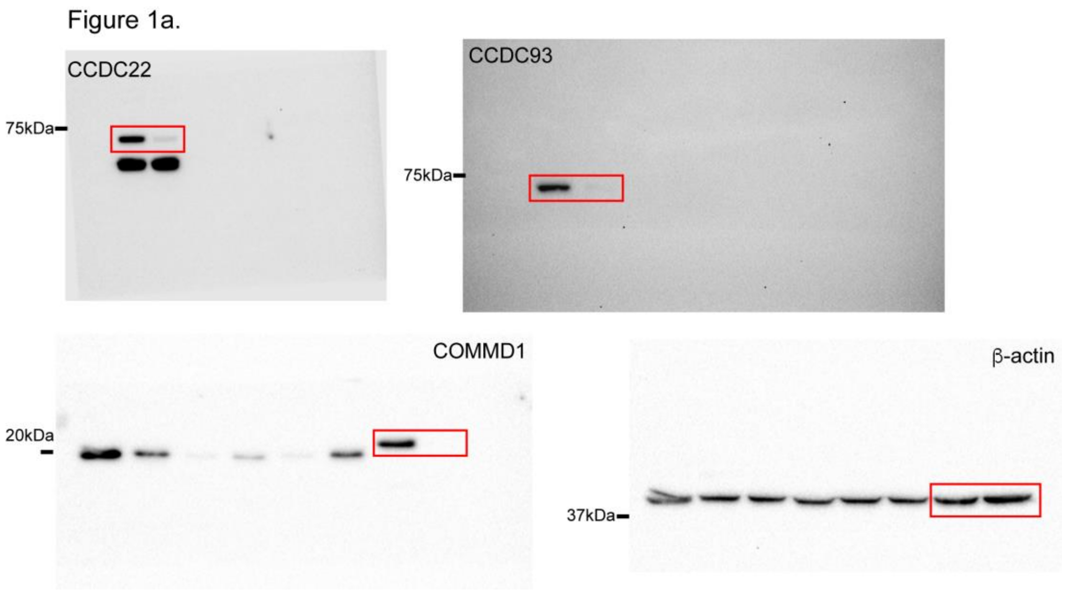

Supplementary figure 12. Full scans

Supplementary Figure 12.

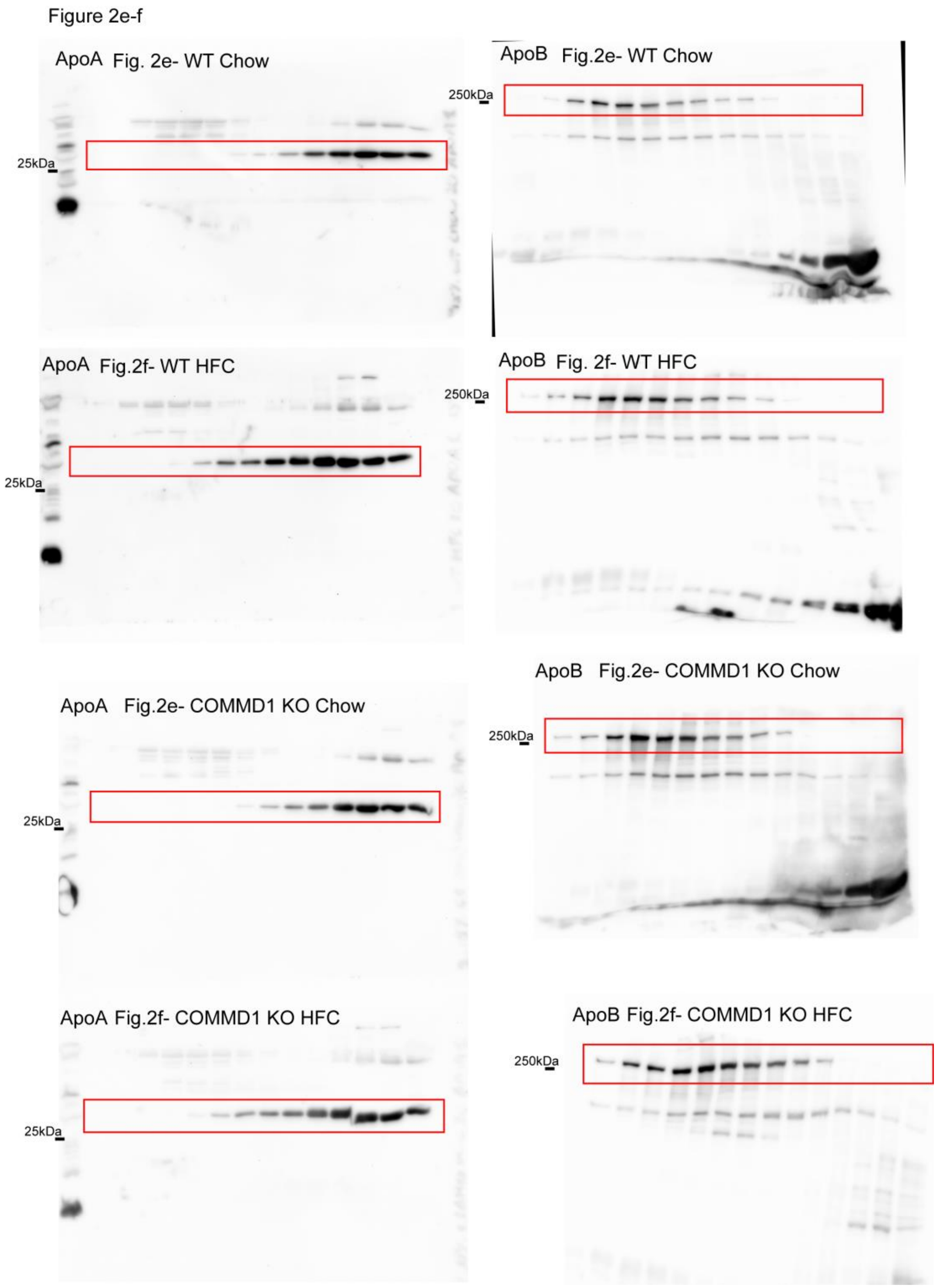

Supplementary figure 12. Continued

Supplementary Figure 12.

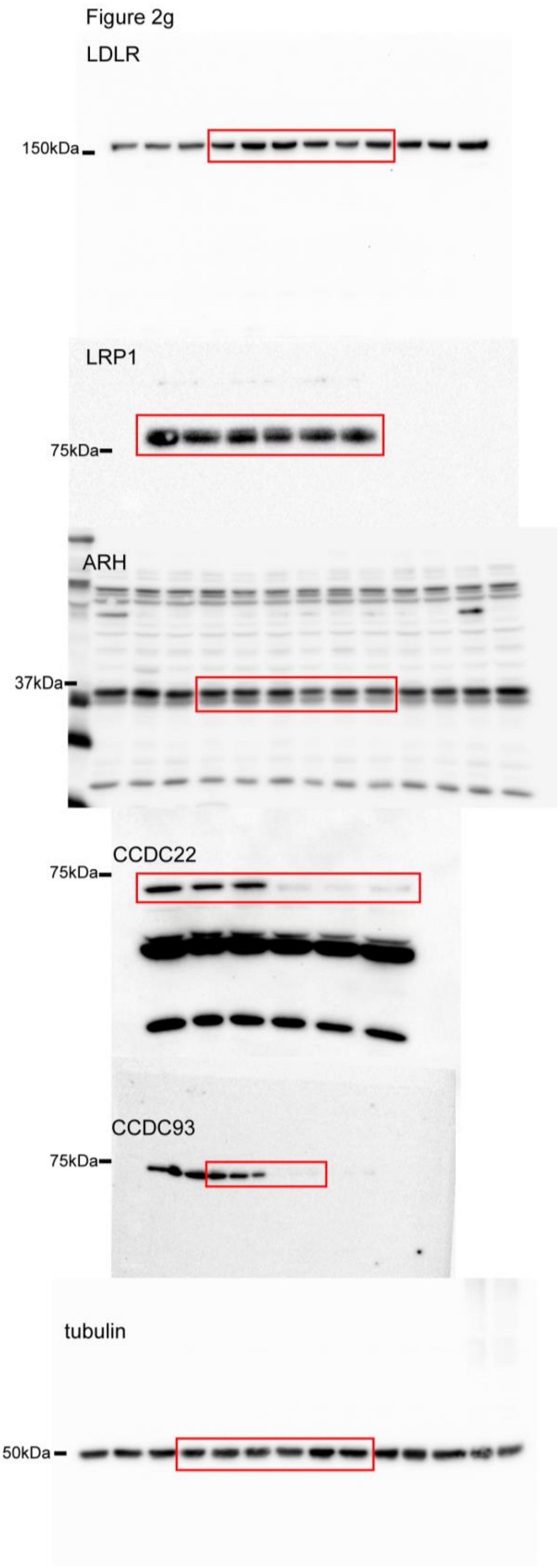

Supplementary figure 12. Continued

Supplementary Figure 12.

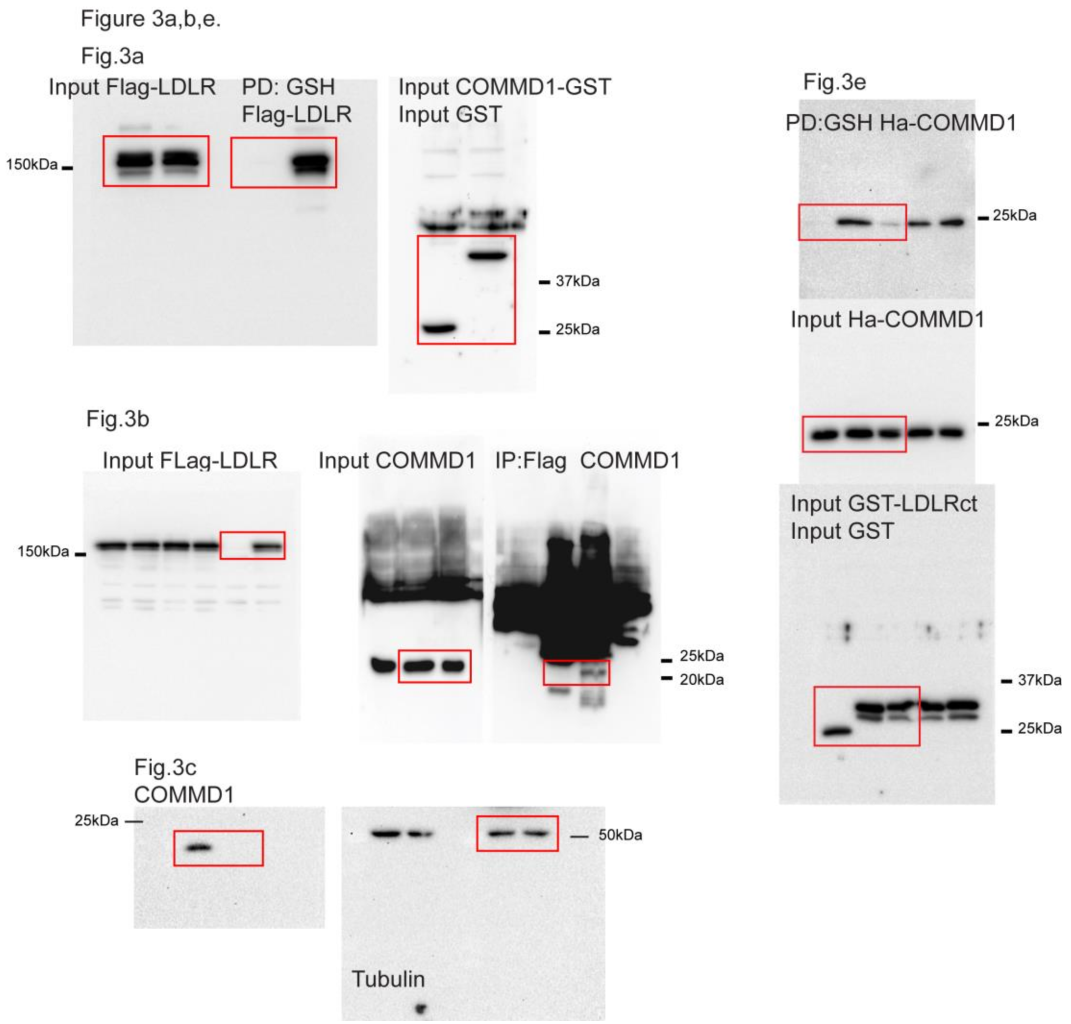

Supplementary figure 12. Continued

Supplementary Figure 12.

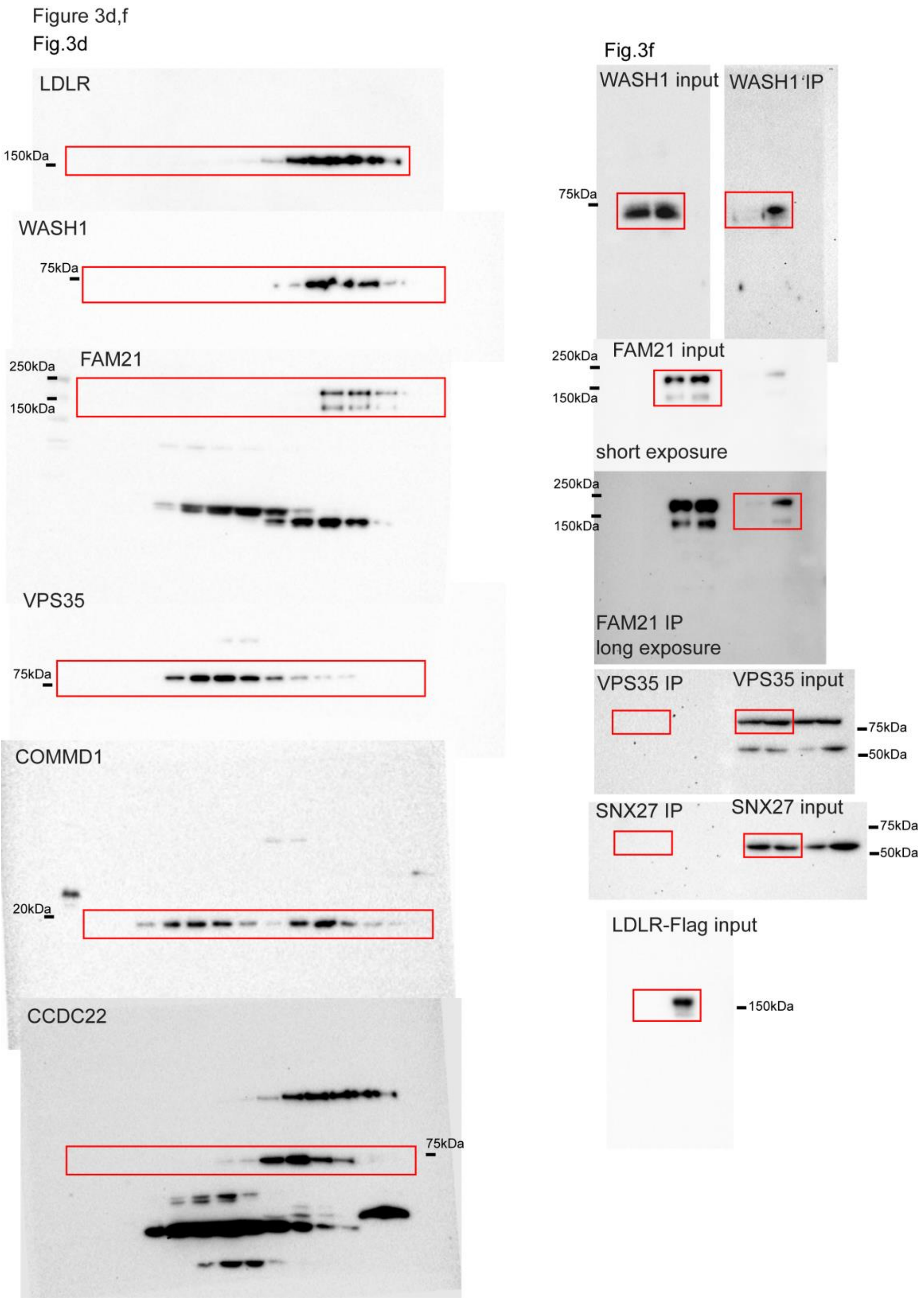

Supplementary figure 12. Continued

Supplementary Figure 12.

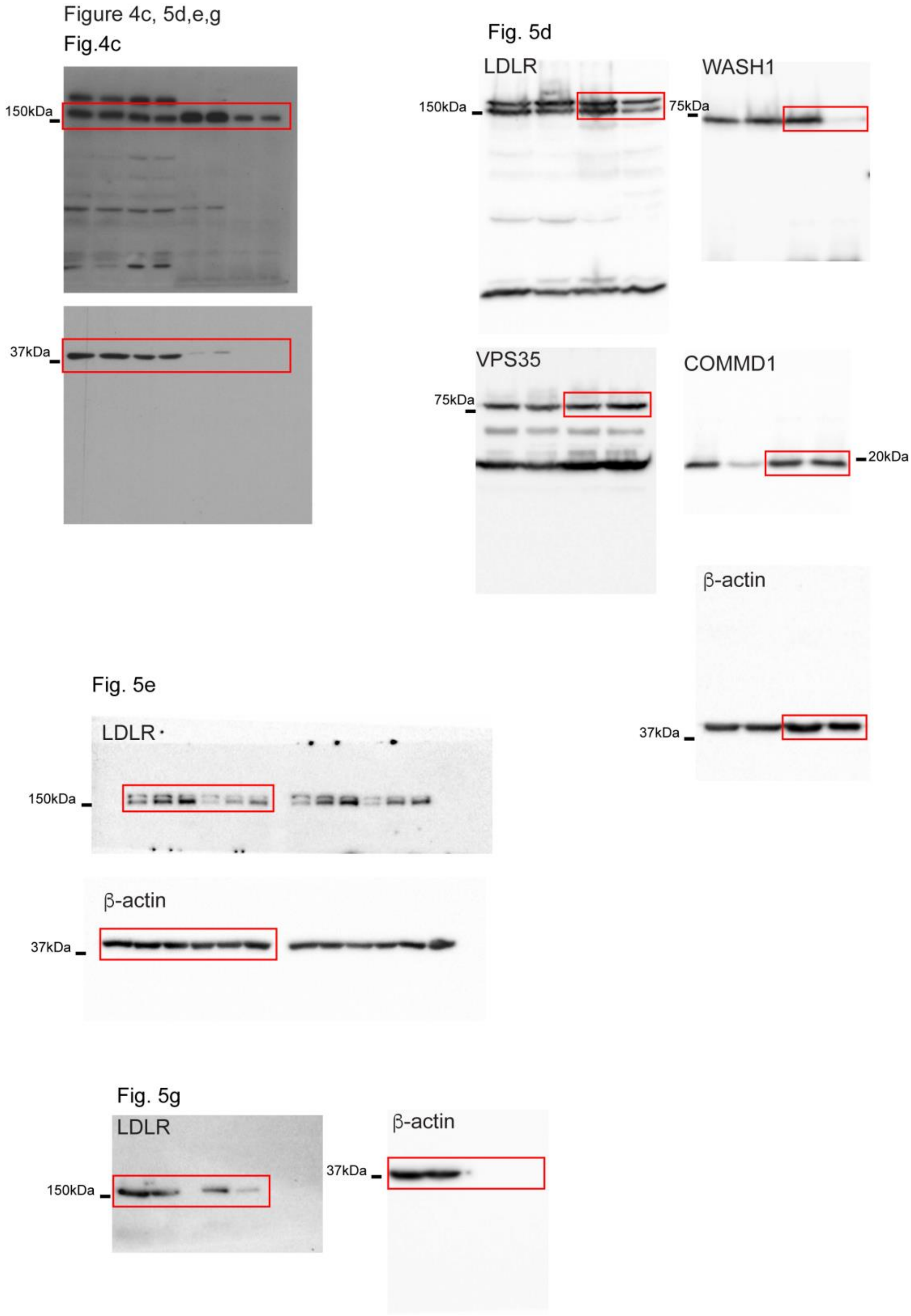

Supplementary figure 12. Continued

Supplementary Figure 12.

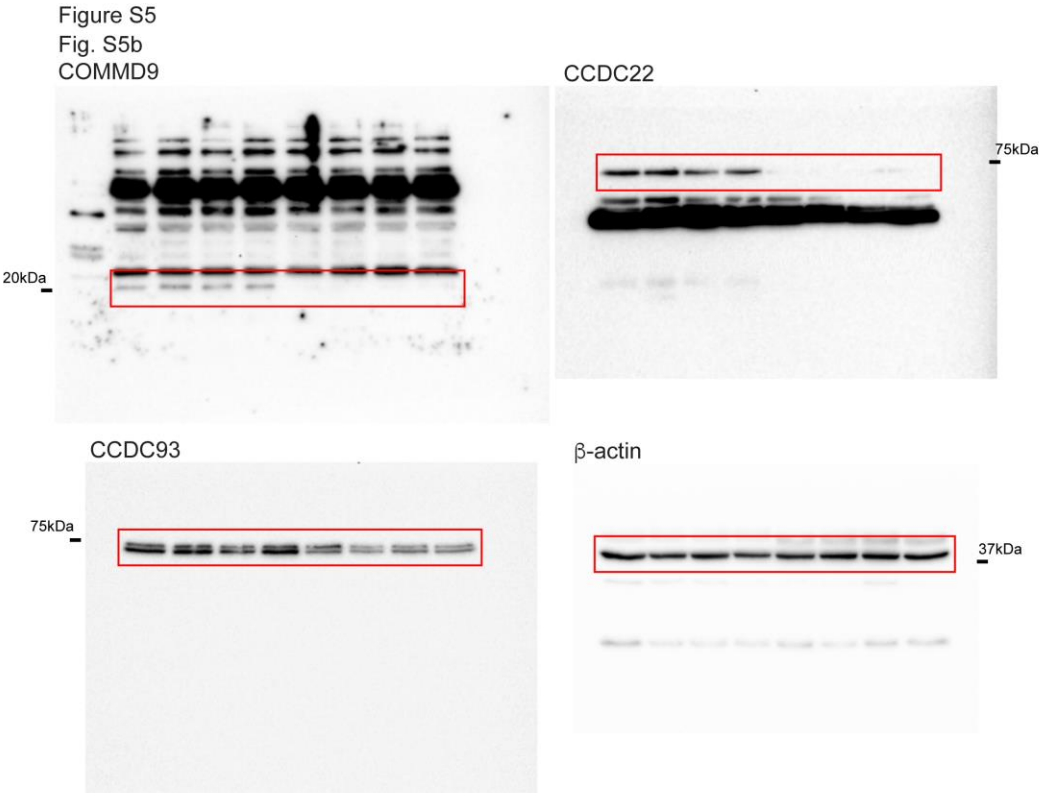

Supplementary figure 12. Continued

Supplementary Figure 12.

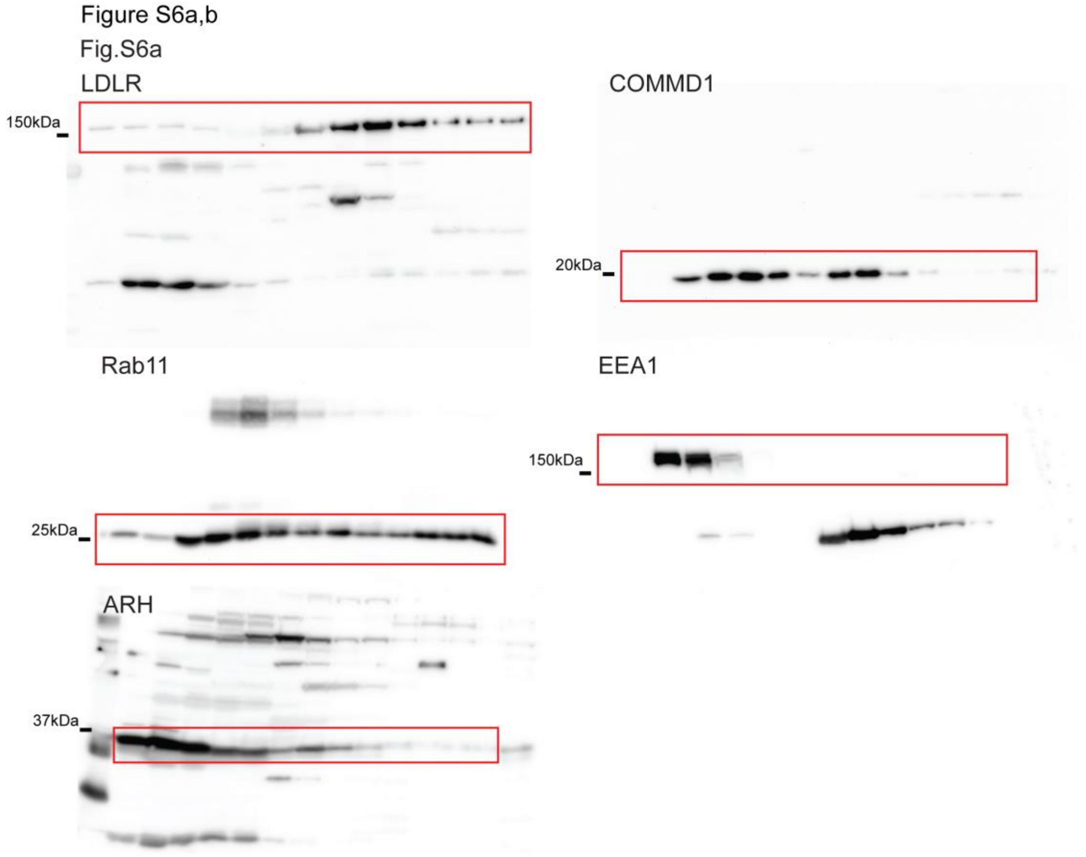

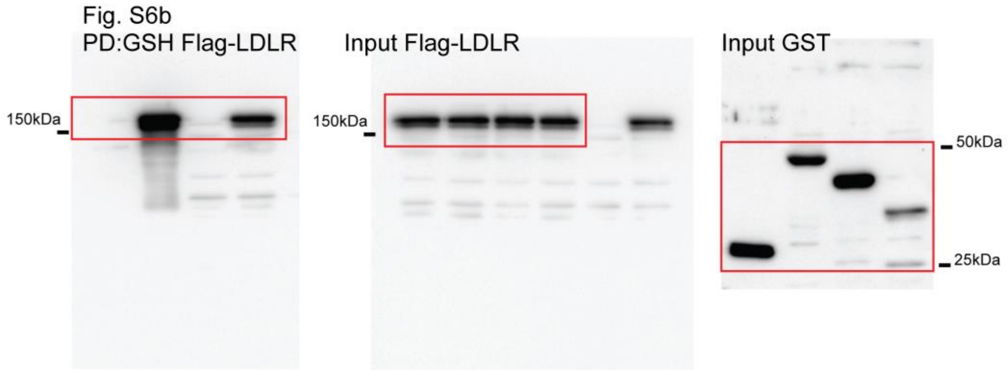

Supplementary figure 12. Continued

Supplementary Figure 12.

Fig.S7b

IP:FAM21 SNX27-GFP   input SNX27-GFP

IP:FAM21 FAM21   input FAM21

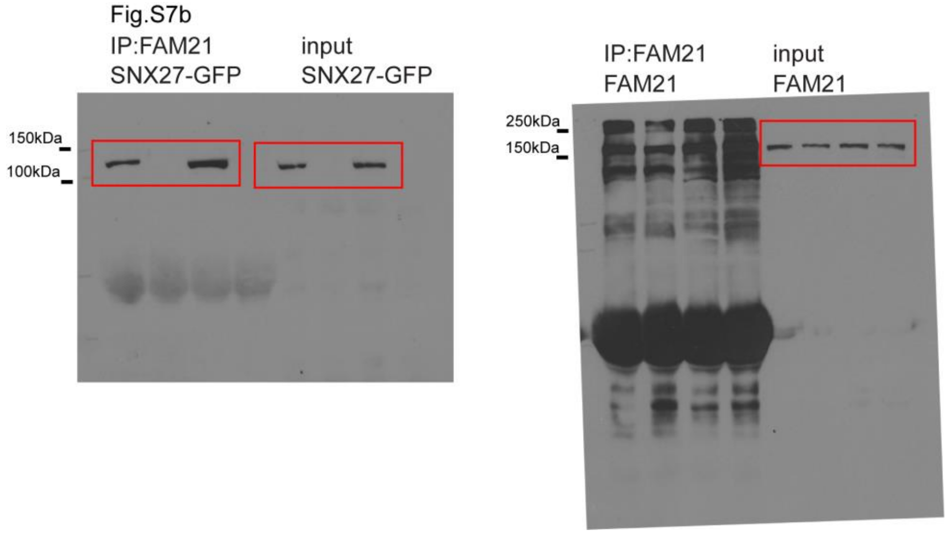

Figure S9

Fig. S9b

LDLR

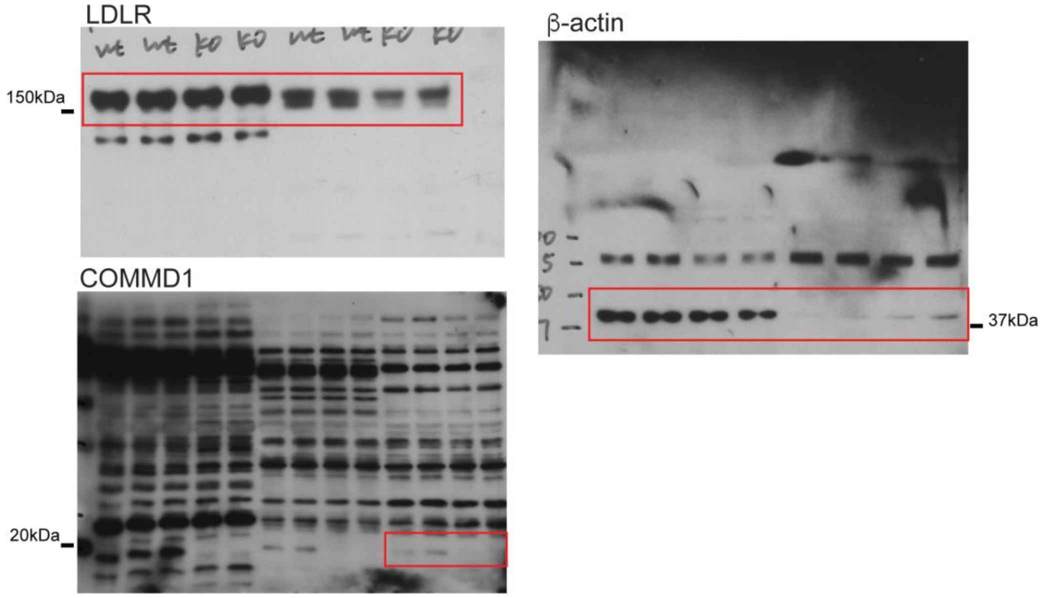

Supplementary figure 12. Continued

Supplementary Table 1.

Parameters of WT and *Commd1*$^{\Delta Hep}$ mice fed chow diet. Values ±s.e.m.

**Age**	28 weeks	28 weeks	**Significance**
**Genotype**	*WT*	*Commd1*$^{\Delta Hep}$	-
**n**	6	7	-
**BW [g]**	32.73 ± 1.41	33.24 ± 1.13	NS
**Liver TC [μmol/g liver]**	2.41 ± 0.43	2.353 ± 0.21	NS
**Liver TG [μmol/g liver]**	12.12 ± 2.09	13.00 ± 2.34	NS
**Liver Copper [μg/g dry liver]**	84.63 ± 8.93	74.99 ± 8.30	NS

Supplementary Table 2.

Parameters of WT and *Commd1*$^{\Delta Hep}$ mice fed HFC diet. Values ±s.e.m.

**Age**	28 weeks	28 weeks	**Significance**
**Genotype**	*WT*	*Commd1*$^{\Delta Hep}$	-
**n**	8	7	-
**BW [g]**	39.65 ± 1.40	37.13 ± 1.36	NS
**Liver TC [μmol/g liver]**	9.04 ± 1.05	10.85 ± 0.82	NS
**Liver TG [μmol/g liver]**	22.52 ± 2.36	22.10 ± 2.73	NS
**Liver Copper [μg/g dry liver]**	17.52 ± 2.11	23.51 ± 2.21	NS

Supplementary Table 3. qRT-PCR primer sequences.

Gene	Forward 5'→3'	Reverse 5'→3'
Ldlr	CATATGCATCCCCAGTCTTTG	GCAGTGCTCCTCATCTGACTTG
Srb1	TTGGCCTGTTTGTTGGGATG	GGATTCGGGTGTCATGAAGG
Lrp	TCAGACGAGCCTCCAGACTGT	ACAGATGAAGGCAGGGTTGGT
Cd36	GATCGGAACTGTGGGCTCAT	GGTTCCTTCTTCAAGGACAACTTC
Hmgcr	AGCTTGCCCGAATTGTATGTG	TCTGTTGTGAACCATGTGACTTC
Srebp2	CGACGAGATGCTACAGTTTG	GGTAGGAGAGACTTTGACCTG
Abaca1	GGGAAGGACATTCGCTCGG	TTGCTTTTCAGCTTGCTCGG
Abcg5	CTGCATGTGTCCTACAGCGTCA	AGATGCACATAATCTGGCCACTCTC
Abcg8	TCAGTCCAACACTCTGGAGGTCA	ATTTCGGATGCCCAGCTCAC
Cyclophilin A	TTCCTCCTTTCACAGAATTATTCCA	CCGCCAGTGCCATTATGG
Commd9	CCTCCTCTGACAACATCAGC	GGAGGGTTTCTCTCCACAC

Supplementary References:

1. Phillips-Krawczak, C. A. *et al.* COMMD1 is linked to the WASH complex and regulates endosomal trafficking of the copper transporter ATP7A. *Mol Biol Cell* (2014). doi:10.1091/mbc.E14-06-1073

2. Kolanczyk, M. *et al.* Missense variant in CCDC22 causes X-linked recessive intellectual disability with features of Ritscher-Schinzel/3C syndrome. *Eur J Hum Genet* (2014). doi:10.1038/ejhg.2014.109
